# Synthesis, Characterization, and Applications of Magnetic Nanoparticles Featuring Polyzwitterionic Coatings

**DOI:** 10.3390/polym10010091

**Published:** 2018-01-18

**Authors:** Philip Biehl, Moritz von der Lühe, Silvio Dutz, Felix H. Schacher

**Affiliations:** 1 Institute of Organic and Macromolecular Chemistry (IOMC), Friedrich Schiller University Jena, Humboldtstraße 10, 07743 Jena, Germany; philip.biehl@uni-jena.de (P.B.); moritz.von-der-luehe@uni-jena.de (M.v.d.L.); 2 Jena Center for Soft Matter (JCSM), Friedrich Schiller University Jena, Philosophenweg 7, 07743 Jena, Germany; 3 Institute of Biomedical Engineering and Informatics, Technische Universität Ilmenau, 98693 Ilmenau, Germany

**Keywords:** hybrid materials, magnetic nanoparticles, polyzwitterions, polyampholytes

## Abstract

Throughout the last decades, magnetic nanoparticles (MNP) have gained tremendous interest in different fields of applications like biomedicine (e.g., magnetic resonance imaging (MRI), drug delivery, hyperthermia), but also more technical applications (e.g., catalysis, waste water treatment) have been pursued. Different surfactants and polymers are extensively used for surface coating of MNP to passivate the surface and avoid or decrease agglomeration, decrease or modulate biomolecule absorption, and in most cases increase dispersion stability. For this purpose, electrostatic or steric repulsion can be exploited and, in that regard, surface charge is the most important (hybrid) particle property. Therefore, polyelectrolytes are of great interest for nanoparticle coating, as they are able to stabilize the particles in dispersion by electrostatic repulsion due to their high charge densities. In this review article, we focus on polyzwitterions as a subclass of polyelectrolytes and their use as coating materials for MNP. In the context of biomedical applications, polyzwitterions are widely used as they exhibit antifouling properties and thus can lead to minimized protein adsorption and also long circulation times.

## 1. Introduction and Scope

For decades, magnetic nanoparticles (MNP) have been in focus within a range of scientific disciplines as they show high potential in a variety of different application fields, ranging from chemistry, biology, medicine to physics. One unifying aspect herein are surface properties of such nanomaterials. To date, there have been several reviews focusing on surface modifications of nanomaterials with polyelectrolytes, and most of them have focused on biomedical applications of these materials [1,2,3,4,5,6,7]. However, to our knowledge the only example specifically focusing on zwitterionic coating materials for nanomaterials was written by García et al. and here the central aspect is the behavior under in vivo conditions [4]. Within this review article, we therefore focus on the preparation and characterization of MNP featuring zwitterionic coating materials as they open up an interesting area of bio-repellent, pH responsive, and dispersion-stable hybrid materials. The magnetic core enables the selective separation of these particles for analytical issues and external magnetic fields can be used for biomedical applications like hyperthermia and drug targeting. This review aims to serve as a guide for various synthetic strategies for immobilizing polyzwitterions at the surface of magnetic nanoparticles which have been explored during the last decade and is structured as follows: we begin with a section on different magnetic core materials, followed by the synthesis of polyzwitterions, suitable methods for nanoparticle coating, and finally we discuss important characterization methods for such hybrid materials. Throughout the different chapters, we also showcase potential application fields.

## 2. The Core: Materials for Magnetic Nanoparticles

All chemical elements or compounds of our planet show under certain conditions different magnetic effects. Since we focus herein on magnetic nanoparticles for medical and technical applications, we concentrate on materials with ferro- or ferrimagnetic, superparamagnetic, and superferrimagnetic behavior at room temperature. In that regard, three classes of materials exist.

***Metals***—The only metallic elements showing ferromagnetism at room temperature are iron, cobalt, and nickel. The preparation of nanoparticles hereof is possible and such materials show promising magnetic behavior for medical applications [8,9,10,11,12,13]. Since such nanoparticles show a strong oxidation tendency to non-magnetic oxides (e.g., antiferromagnetic FeO, CoO, NiO), an oxidation-protective layer is necessary. Due to this fact, and also the toxicity of Ni and Co, metallic nanoparticles play only a minor role regarding their applications in medicine [14].

***Alloys***—The second group of ferromagnetic materials are the ferromagnetic alloys, e.g., CoPt, FePt, FeNi, or FeCo. The preparation of magnetic nanoparticles consisting of ferromagnetic alloys is described in the literature by several groups [15,16,17]. Up to now, none of those nanostructures has found access in medical applications mainly due to two facts: First, some of the ferromagnetic alloys (e.g., AlNiCo, CoPt, FeCoCr) show a hard-magnetic behavior (a remnant magnetization and coercivity), leading to potential agglomeration of the particles due to the remanence, and exposing the patient to the risk of vessel embolism. Second, most of the alloys with promising magnetic behavior contain toxic components (e.g., Ni or Co) which inhibit the application of such materials in the human body.

***Oxides***—The group of magnetic oxide materials can be divided into mixed oxides with different crystal structures (e.g., the magnetic garnets and the ferrites) as well as the pure metallic oxides. Since the saturation magnetization of all garnets is very low, these materials are not suitable for application in medicine. Depending on their composition, the ferrites show soft- or hard-magnetic behavior. Despite some groups having found promising magnetic properties of soft-magnetic ferrites for certain medical applications only very few studies can be found in the literature [18,19,20,21]. Representative hard-magnetic ferrites with promising magnetic behavior for medical application are barium-, strontium- or cobalt-ferrite. Since cobalt-ferrite (CoFe_2_O_4_) shows less toxic effects than Ba-or Sr-ferrite, nanoparticles of this material find increasing application for medical purposes, e.g., for magnetic hyperthermia as minimal invasive tumor treatment and for lab-on-a-chip applications in diagnostics [22,23,24]. The promising magnetic properties of cobalt-ferrite can be tuned by variation of the Co/Fe-ratio and thus this material will play a major role in the future in our opinion. As Ni and Co form no oxides showing ferromagnetism at room temperature, only iron has to be considered in this case. Here, mainly four different oxides have to be mentioned: iron(III) oxide (Fe_2_O_3_) and iron(II,III) oxide (Fe_3_O_4_), as well as the rather unstable iron(II) oxide (FeO) and iron(I) oxide (Fe_2_O). From Fe_2_O_3_ several phases exist, e.g., α-, β-, γ-, or ε-Fe_2_O_3_, which all show different magnetic behavior. Of the iron oxides only maghemite (γ-Fe_2_O_3_) and magnetite (Fe_3_O_4_) show ferromagnetic behavior or, more precisely, ferrimagnetism due to the spinell structure (a subtype of the cubic lattice). A comprehensive work on the nature of iron oxides and their properties is given by Schwertmann [25].

The preparation of iron oxide magnetic nanoparticles was described by Khallafalla [26] and Massart [27] in 1980 for the first time. After that, a lot of different preparation routes were developed and such MNP show promising magnetic properties for different biomedical applications [28,29].

### 2.1. Magnetic Properties of Magnetic Nanoparticles

Beside other parameters like magnetic anisotropy or shape, the magnetic behavior of magnetic particles is determined by the particle size. For macroscopic particles in the size range of µm and above, several areas of homogeneous magnetization are formed. These so-called magnetic domains are separated by Bloch walls [30,31]. Due to this domain formation, the magnetic stray field of the particle is minimized and the domain formation in the absence of an external magnetic field is energetically favorable [32] compared to a homogeneously magnetized particle. The magnetization directions of all domains in the particle are statistically oriented, which leads to a compensation of all magnetic moments within the particle, resulting in no external magnetization of the particle without an external magnetic field.

With decreasing dimensions of the magnetic particle, the relative proportion of wall energy to that of the entire particle energy increases. Due to energetic reasons, no magnetic domains are formed below a critical particle size and the whole particle shows a spontaneous magnetization in one direction. The direction of the magnetization of these so-called single domain particles is determined by the crystal lattice of the particle and is named “the easy axis”. The critical size for the formation of single domain particles is given by the material specific magnetic anisotropy K and the form factor (ratio of particle length in different directions related to the magnetic field) of the particle [33]. For cubic and spherical particles made of magnetite, the theoretical upper limit for the formation single domain particles is about 80 nm [34,35], which was confirmed experimentally by Dutz et al. [36].

A further decrease of the particle size leads to a decrease of the magnetic anisotropy energy of the particles. In this case a certain probability exists, that for finite temperatures the thermal energy exceeds the anisotropy energy due to thermic variations and the particle spontaneously changes the orientation of magnetization [37]. This leads to a thermally induced temporal attenuation (relaxation) of the remnant magnetization *M_R_* following Equation (1):

*M_R_*(*t*) = *M_R_*(*t* = 0) × *e^−t/τN^* (1)

The so-called Neel relaxation time *τ_N_*, after which *M_R_* reaches a value close to zero, can be estimated from the ratio of the anisotropy energy (*K* × *V*) to the thermal energy (*k* × *T*) with the Boltzmann constant *k* and the temperature *T* following Equation (2) where *τ*_0_ is the minimum natural relaxation time of 10^−9^ s:

*τ_N_* = *τ*_0_ × *e*^(*K* × *V*)/(*k* × *T*)^ (2)

Hence, the magnetic behavior of very small particles depends strongly on the relation of measurement time *t_M_* and Neel relaxation time *τ_N_*. If *t_M_* << *τ_N_*, there is not enough time for relaxation processes and the particles show a stable hysteretic behavior. If *t_M_* > *τ_N_*, the Neel relaxation occurs, leading to attenuation of *M_R_* and thus no coercivity can be observed. This phenomenon is called superparamagnetism. Superparamagnetic particles show no coercivity and remnant magnetization in quasi-static measurements (e.g., vibrating sample magnetometry) but a pronounced hysteresis when exposed to a high frequency alternating magnetic field. In other words, for predetermined magnetic field parameters (frequency and field strength), it depends on the particle size whether the particles show any hysteresis or not.

A special case of magnetism can occur if small superparamagnetic particles form a larger cluster. In the absence of an external magnetic field these clusters show superparamagnetic behavior with no remnant magnetization or coercivity. If the particles are exposed to an external field, depending on the strength of the particle interactions, a collective magnetism may result and the clusters show ferrimagnetic behavior with an observable hysteresis. This so-called superferrimagnetism is typical for magnetic multicore particles [38,39,40] and such particles show very promising properties for medical applications [38,41,42,43].

From the considerations above it becomes obvious, that the particle size plays a crucial role for the magnetic behavior of magnetic nanoparticles. Besides the size, also the size distribution is an important factor for the resulting magnetic properties, which will not be treated in detail in this review. Detailed discussions on the theory of size distribution influence can be found by Hergt et al. [44] whereas Müller et al. showed the influence in experiments [45].

### 2.2. Preparation of Magnetic Nanoparticles

The following section briefly covers the main preparation routes for magnetic nanoparticles. Detailed information can be found in excellent reviews on this topic [46,47,48,49]. Magnetic nanoparticles are obtained by three different preparation routes.

(i) biomineralization
(ii) physical methods
(iii) chemical methods
  (i) By means of *biomineralization* some living organisms prepare magnetic particles for use for their sense of direction [50]. For example, magnetotactic bacteria are capable of preparing magnetosomes (protein coated nanosized crystals of magnetic iron oxide). The bacteria use the particles as a compass to find their preferred habitat in anaerobic areas at the bottom of the sea [51]. Under anaerobic synthesis conditions in the lab, which are similar to the conditions of their habitat, uniform particles of 20 to 45 nm core diameter may be produced [52,53,54]. Despite the fact that magnetosomes show excellent magnetic properties for medical application (especially hyperthermia) [55,56], they have found no application in medicine until now due to their bacterial protein coating. Current recent research on magnetosomes focuses on elucidation and optimization of the biomineralization process [57,58] with the aim to develop wet chemical preparation routines which emulate the biologic process, thus providing MNP with similar magnetic behavior.
  (ii) The *physical methods* can be divided into “top down” and “bottom up” procedures. Top down methods are based on the size reduction of macroscopic magnetic materials to the nanometer range, e.g., by means of milling. A major drawback of these methods is the difficulty of adjusting the desired particle size and shape [59]. Furthermore, the milling procedure leads to lattice defects that cause deviations in the magnetic properties compared to regular particles of the same size [60]. Bottom up methods use the condensation of nanoparticles from either a liquid or gaseous phase. A promising bottom up method for the synthesis of MNP powders is laser evaporation. Starting materials are coarse metal oxide powders of a few µm sized particles, which are evaporated by means of a laser. As a result of the steep temperature gradient outside of the evaporation zone, a very fast condensation and nucleation takes place from the gas phase and nanoparticles are formed [61,62].The resulting mean particle sizes (20 to 50 nm) and magnetic phase are tuned by laser power and composition of the atmosphere in the evaporation chamber [63].
  (iii) The *chemical methods* provide a multitude of different bottom up synthesis routes for the preparation of MNP, from which the most prominent will be described shortly.

The *co-precipitation* synthesis procedure is a very simple method for the preparation of MNP. Most scientific work uses aqueous media for precipitation. Very often, the magnetic iron oxides are prepared by means of a co-precipitation from aqueous Fe^2+^ and Fe^3+^ salt solutions, to which a base is added. Magnetic phase and particle size can be tuned by variation of iron salts, Fe^2+^/Fe^3+^ ratio, temperature, pH, and the type of base used. Pioneering work on this preparation route was performed by Khallafalla and Reimers [26] and Massart [27]. For this method, particles are in the superparamagnetic size range from 5 to 15 nm and the obtained size distribution is relatively broad. By varying the reaction conditions, the size can be increased to up to 40 nm. In this size range, the particles show single domain ferrimagnetic behavior. Different modifications of this method were reported over recent years. Upon applying high pressure homogenization during precipitation [64] or using slower reaction conditions [39], superferrimagnetic clusters of single crystals of 10 to 15 nm are formed, which show very promising magnetic properties for medical applications [42,65]. Furthermore, size control of the resulting magnetite nanoparticles could also be shown by reactions carried out at high temperatures [66]. Co-precipitation is also used for the preparation of ferrites, e.g., cobalt ferrite by replacing a part of the Fe^2+^ by Co^2+^ in the starting solutions [67].

The *thermal decomposition* of organometallic compounds (non-magnetic precursors) in boiling organic solvents is another promising way for MNP preparation and the resulting particles show a very narrow size distribution. Usually iron carbonyls or iron acetylacetonates are used as non-magnetic precursors and oleic acid or fatty acids serve as surfactants. By variation of the proportion of precursors to the starting agents (surfactants and solvents), the size and morphology of the resulting particles can be controlled. Thermal decomposition of non-magnetic precursors leads to pure iron (metal). Afterwards, in a further step these metal particles are oxidized to iron oxide by mild heating under oxidative conditions. A simple one-step route to prepare magnetite particles is given by the thermal decomposition of precursors with cationic iron centers (e.g., Fe(acac)_3_). Pioneering work in the preparation of iron oxide by thermal decomposition was performed by Hyeon et al. [68] and Park et al. [69] who prepared nearly monodisperse particles of about 13 nm. The well-known method of Hyeon and Park was modified by several groups and MNP in size of up to 30 nm with nearly monodisperse size distribution were obtained.

*Micro-emulsion synthesis* is a two-phase method for the production of nearly monodisperse MNP. For this purpose, a water-in-oil microemulsion is prepared by dispersion of nanosized water droplets (10–50 nm) in an oil phase, stabilized by surfactant molecules at the water/oil interface [70]. Since these droplets are used as micro-reaction vessels, the distance for diffusion and thus the nucleation and growth of particles is limited, which results in very uniform particles [71]. Due to their narrow size distribution, MNP from the microemulsion synthesis show magnetic properties promising for medical applications [72].

*Hydrothermal synthesis* performed in aqueous media at temperatures above 200 °C is realized in autoclaves at pressures above 2000 psi. This route exploits the ability of water to hydrolyze and dehydrate metal salts at high temperatures. Due to the low solubility of the obtained metal oxide particles in water at such temperatures [73,74], a precipitation takes place and by variation of concentration, temperature, and autoclaving time, particle size and morphology can be controlled [75,76]. Longer autoclaving time leads to increasing particle size, but also broader size distributions. Sizes typically are in the range from 10 to 50 nm and for short autoclaving times, monodisperse particles can be obtained [76].

The *polyol synthesis* bases on the oxidative alkaline hydrolysis of Fe^2+^ and Fe^3+^ salts in a polyol mixture (e.g., poly(ethylene glycol) (PEG)/diethylene glycol or *N*-methyldiethanolamine). Size and structure of the resulting MNP can be tuned by either reaction conditions or the employed solvents [77]. Despite the fact that the particles are not monodisperse in size, they show interesting magnetic behavior for application in hyperthermia due to their special morphology. So called “flower-shaped MNP” can be synthesized by this procedure under certain reaction conditions [77] which show excellent heating performance for hyperthermia [43]. Similar to co-precipitated clusters, these particles exhibit a multicore structure and consist of single cores of about 8 to 10 nm. These cores form clusters of about 30 nm and show very promising properties for hyperthermia as shown before [42].

Other preparation routes for magnetic nanoparticles, which are not demonstrated here in this article because the resulting particles are not of high interest for medical applications, are Glass Crystallization [78], Spray and Laser Pyrolysis [79], Sonolysis [48,79], Microwave Irradiation Synthesis [79], and Sol-gel Reactions [80].

### 2.3. Recent Developments in the Synthesis of Magnetic Nanoparticles

Over the past 10 years, the major aim of magnetic nanoparticle preparation was to develop strategies for a versatile and robust protocol for the synthesis of tailor-made samples. Due to the high diversity of the required magnetic properties of the particles for the different applications outlined above and below, several structural parameters (e.g., size and size distribution) have to be tuned. For example, medical applications benefit in three ways from magnetic particles. First, magnetic particles can be manipulated mechanically by an external magnetic field (gradient), resulting in a rotation or attraction of the MNP which can find application in magnetic drug targeting [81,82]. Second, due to their magnetic moment, MNP are a source of a magnetic stray field, which can be detected by appropriate sensors and might find application in medical imaging [83]. Finally, if MNP are exposed to an alternating magnetic field, the particles are heated up due to reversal magnetization losses and the generated heat can be used for therapeutical applications, e.g., hyperthermia as an example for minimal invasive cancer therapy [28,84].

To obtain MNP which show promising magnetic behavior for mechanical manipulation, MNP with a high magnetic moment are needed and quite often this is translated into a large particle volume. Several groups obtained different strategies for the preparation of so called large single domain particles (LSDP) [85,86,87,88,89,90]. Despite the fact that the steric stabilization of such large particles is challenging (due to the strong tendency to form agglomerates) sedimentation stable dispersions of large single domain particles exist [86,91]. A possible solution for the challenging stabilization of LSDP is the use of Co-ferrites. They show magnetic properties similar to that of LSDP but much smaller diameters of about 10 to 15 nm [92], which enable sufficient steric stabilization.

The ideal MNP for application in medical imaging need a magnetic behavior, which is described by a high initial susceptibility. The preparation of such particles is challenging since the size of the particles has to be exactly adjusted and the particles need a very narrow size distribution. Usually the thermal decomposition method is the most promising way for the preparation of such particles. Krishnan et al. prepared particles of 25 nm size which exhibited a very narrow size distribution, and so far showed the best performance for magnetic particle imaging [93]. Similar preparation routes to obtain MNP of well-defined size and narrow size distribution are described by other groups in the literature.

For magnetic heating applications (medical or technical) the MNP have to be optimized in a way that reversal magnetization losses are as high as possible for given magnetic field parameters. To reach this aim several strategies exist. For the application of relatively low magnetic fields (<10 kA/m), small MNP with a size of about 10 nm and resulting superparamagnetic behavior are the most promising candidates, which mostly consist of iron oxide. If higher magnetic fields (10 to 30 kA/m) are acceptable, larger ferrimagnetic particles show much better heating performance than superparamagnetic examples. This is due to different mechanisms of internal reversal of magnetization in ferrimagnetic and superparamagnetic samples by means of hysteresis or Neel relaxation, respectively. Such magnetic behavior can be obtained from single-domain iron oxide particles with larger diameter as described above as LSDP for drug targeting or by Co-ferrites which combine a small particle diameter with a defined hysteretic behavior [22]. Also for heating applications, the particle size distribution plays a crucial role in obtaining the ideal heating performance. Usually a narrow size distribution is preferred but for some combinations of particle size, magnetic field frequency, and strength a higher heating performance also for MNP featuring a broader size distribution has been reported [45,94].

Over the past years two different particle types were developed which show a magnetic behavior that cannot be achieved by the classical single-core particles. One example is again the so called superferrimagnetic multicore-particles. This particle type consist of primary cores in the range of 10 nm with superparamagnetic behavior which form clusters of about 50 nm or larger [39,41,43,95]. Due to the statistical orientation of the easy axis of the single cores within the clusters, the resulting magnetization without any external field is relatively low in comparison to single core particles of comparable size. Due to this fact these large particles show a relatively weak remnant magnetization and also only a very low agglomeration tendency. Therefore, such particles are relatively stable against sedimentation, which is a general requirement for medical applications. If these particles are exposed to an external magnetic field, the clusters show a coercivity higher than that observed for the size of the constituting primary particles but lower than reported for single-domain particles in the size regime of the clusters. Up to now there is no existing theory capable of completely describing the magnetic behavior of these particles, but experimental investigations revealed promising results in different application areas [38,41,43]. Exchange-coupled magnetic nanoparticles are the second novel particle type [96,97]. These particles benefit from the exchange coupling between a magnetically hard core (e.g., Co-ferrite) and a magnetically soft shell (e.g., Mn-ferrite). This interaction enables tuning of the magnetic properties of the nanoparticle and the maximization of the reversal magnetization losses, which renders these particles very interesting for heating applications [96,98,99]. Typically, at first the hard magnetic core is prepared and then the soft magnetic shell is deposited on the core surface. By changing the material combinations and ratio of core and shell size the resulting magnetic properties can be tuned.

## 3. The Shell: Polyzwitterions

**Figure 1:**
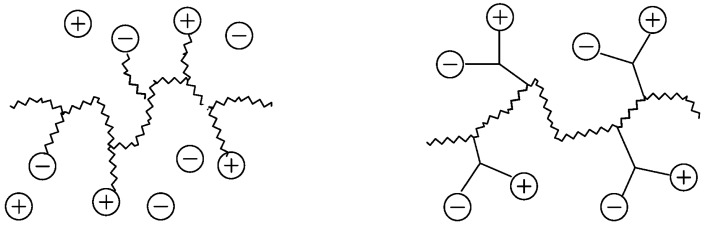
Polyampholyte (**left**) bearing both negative and positive charges statistically distributed along the polymer backbone, and (**right**) polyzwitterion, bearing both charges in every repeating unit. Reprinted from [101].

In the field of polyelectrolytes, polyzwitterions have gained significant interest over recent years due to their tunability concerning charge density, net charge, and as anti-fouling coatings of different surfaces. Polyzwitterions are defined by IUPAC as polyelectrolytes that, unlike polyampholytes, carry both cationic and anionic groups in every repeating unit [100]. Nevertheless, in the literature the term polyzwitterion is sometimes mixed up with that of polyampholytes (Figure 1). In this review, we focus on polyzwitterionic materials as coatings on magnetic nanoparticles.

As mentioned above, polyzwitterions are of great interest as coating materials especially for biomedical applications, as they are reported to inhibit non-specific protein adsorption [102,103]. For example, betaines like poly(carboxybetaine acrylamide) (pCBAA) [104], poly(sulfobetaine methacrylate) (pSBMA) [105], or poly(carboxybetaine methacrylate) (pCBMA) [106] show ultralow biofouling, which was attributed to their strong hydration capacity caused by electrostatic interactions between the zwitterionic moieties and water [107]. Furthermore, the attachment of polyzwitterions onto MNPs is not accompanied by a huge increase in their hydrodynamic radii, which is of great interest as a specific size between 30 and 200 nm is targeted for MNPs to achieve longer circulation times [108], and ideal properties for passive accumulation within tumor tissue [109]. At the moment, poly(ethylene glycol) (PEG) is still the most commonly used polymeric coating if the minimization of unspecific protein adsorption is targeted [110,111], with the major drawback that these systems tend to undergo oxidative degradation. Additionally, these polyether compounds exhibit the so-called “stealth” effect preventing a response of the immune system [112]. In contrast to this, zwitterionic moieties are often found in biological systems as is the case for different phospholipids, which build up the main component of biomembranes [113], featuring zwitterionic, hydrophilic head groups (phosphatidyl-cholin, -Ethanolamin, -Serin) and enzymes which consist of polypeptides. Compared to other polyions, polyzwitterions exhibit long circulation times [114,115,116], whereas polycations usually show unspecific and fast binding to cell membranes and might cause cytotoxic side effects [117]. Nevertheless, binding to cell membranes is in general possible also possible for polyzwitterions without the challenge of overcoming repulsive forces from the (in general) negatively charged cell surface.

**Figure 2:**
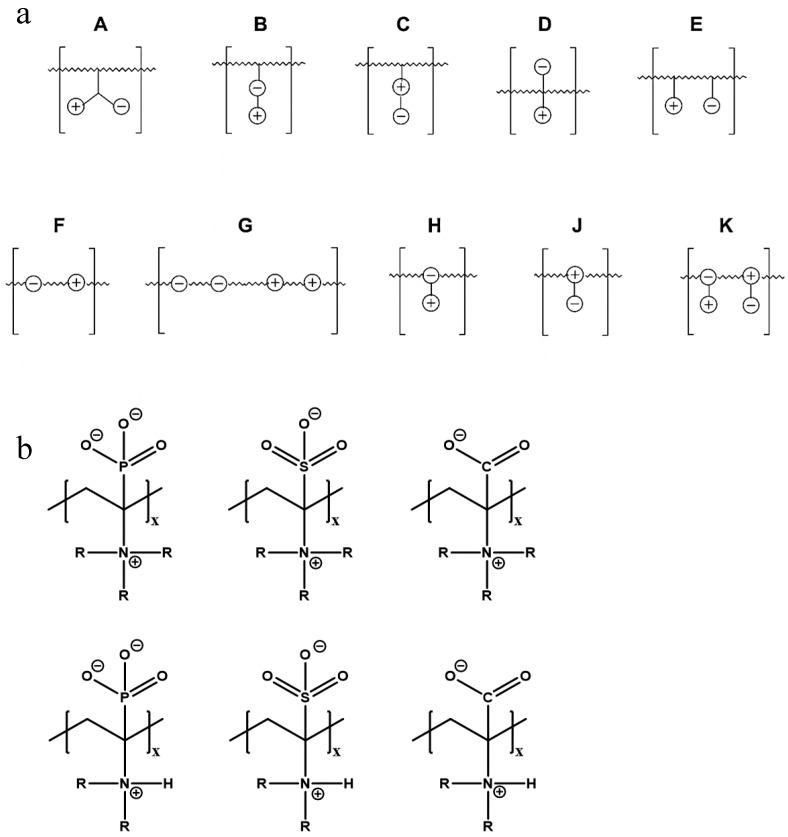
(**a**) Different arrangements of the functional groups in polyzwitterionic chains, reprinted from [101], and (**b**) commonly employed zwitterionic repeating units in polymeric materials.

The given definition of polyzwitterions as polyelectrolytes, which carry both anionic and cationic functionalities in every repeating unit still allows several possibilities for the implementation of the respective functional groups in the polymer structure (Figure 2). Different synthetic routes to obtain polyzwitterions are discussed in the following chapter. A detailed discussion on the various possibilities and synthetic routes can be found in an excellent recent review [101].

It has been discussed that the implementation of zwitterionic moieties in the side chains (A–D) is often easier than directly within the polymer backbone (E–K). Along the same lines, the functionalization of cationic groups such as ammonium moieties is usually more straightforward if compared to the anionic counterparts, concluding that structure C is the most common polyzwitterion structure found in the literature today. The high amount of ionic groups per monomer unit results in rather high charge densities, whereas the net charge of polyzwitterions remains low over a wide pH range (depending on the nature of the ionic groups) due to the stoichiometric presence of oppositely charged groups. Besides the arrangement of the charged functionalities, their chemical design has a major influence on the properties of the resulting material. For cationic groups, most examples reported rely on amines or their quaternized ammonium analogues. Whilst the charge density of the primary amine depends on the pH value of the surrounding medium, upon quaternization these groups are permanently charged. For negative charges, the employed variety of functional groups is broader. Most common are carboxylates, sulfonates, and phosphates [118,119,120,121], less common examples are phosphonates [122,123,124], phosphinates [123], boronates [125] or sulfonamides [126]. Since sulfuric acids commonly show pKa values <1, the charge density of sulfonates does not depend on the pH (in the range of 1–14), whereas phosphates (pKa = around 2) and carboxylates (pKa between 1 and 5) show pH-dependent charge characteristics (degrees of neutralization). Different combinations of the above mentioned weak and strong functional groups lead to four possible categories of polyzwitterions with the combinations of cation/anion: weak/weak (e.g., primary amine/carboxylic acid), weak/strong (e.g., primary amine/sulfonic acid), strong/weak (e.g., quaternized amine/carboxylic acid), and strong/strong (e.g., quaternized amine/sulfonic acid).

## 4. Coating of Magnetic Nanoparticles

**Figure 3:**
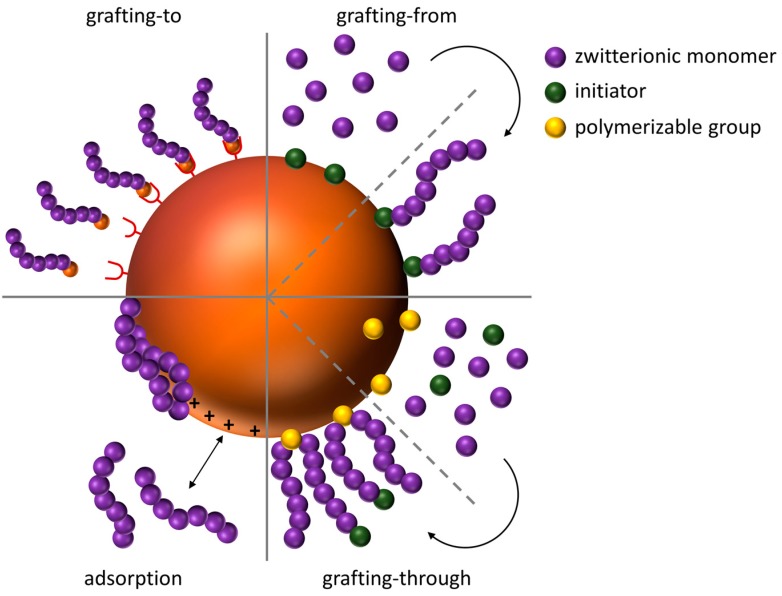
Schematic representation of different grafting methods of polymers to nanoparticle surfaces.

In general, coating procedures for magnetic iron oxide nanoparticles can be divided into adsorptive and covalent techniques. Covalent approaches can be further subdivided into grafting-to, grafting-from, and grafting-through approaches (Figure 3).

For covalent attachment of a polymeric shell, prior functionalization of the nanoparticle surface is necessary. The most prominent example is the synthesis of a thin SiO_2_ shell on the surface which can be prepared using the Stoeber process [127]. If functional silane precursors are used, the resulting SiO_2_ surface exhibits additional functional groups such as amines [128] or thiols [129], which can later on be used for grafting procedures of polyelectrolytes [128]. For grafting-to, the respective polyelectrolyte is functionalized with an appropriate endgroup capable of reacting with the modified nanoparticle surface, whereas in grafting-from approaches, the nanoparticle surface is functionalized with an initiator, followed by subsequent surface-initiated polymerization. Covalent grafting-to can be achieved with polyelectrolytes endcapped with triethoxysilanes, which can be bound to the modified nanoparticle surface (e.g., silica precoating). Grafting-from can be realized by functionalization of the nanoparticle surface with initiators for polymerization, e.g., *N*-(2-aminoethyl)-2-bromo-2-methylpropanamide, which has been used for the Atom Transfer Radical Polymerization (ATRP) of carboxybetaine methacrylate from iron oxide nanoparticles [130]. For grafting-through, polymerizable groups can be introduced—for example by condensation of γ-methacryloxy-propyl-trimethoxysilane (MPS) [131].

**Figure 4:**
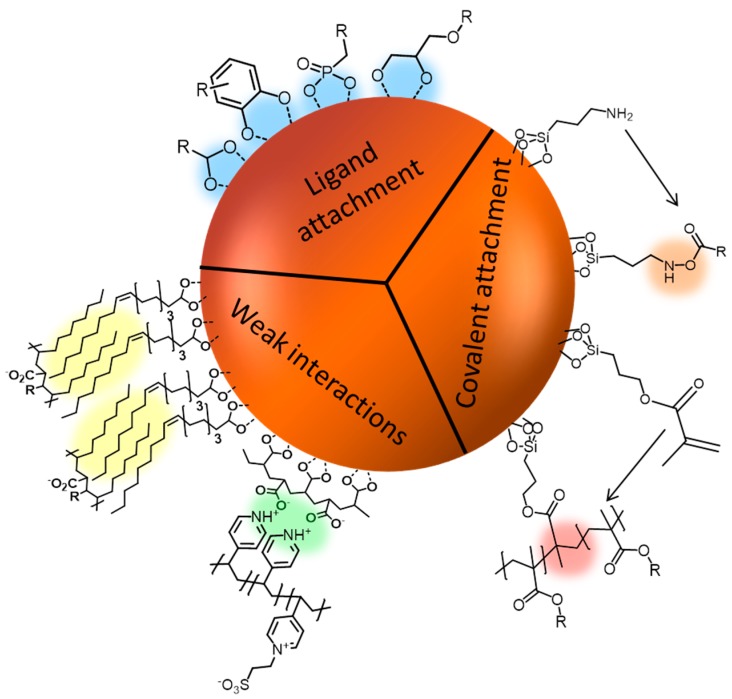
Schematic representation of applied immobilization techniques for polyzwitterions at the surface of magnetic nanoparticles (MNPs).

The most common way to attach polyelectrolytes to nanoparticle surfaces is chemisorption or physisorption by either complexation of iron ions at the surface, electrostatic interactions between polymer and nanoparticle or by exploiting hydrophobic interactions (van-der-Waals forces, Figure 4). Specific examples are the chemisorption of polymers featuring carboxylic acid moieties, as for example shown by von der Lühe et al., who immobilized polydehydroalanine on pristine MNPs [132] or Poimbo Garcia et al. who used MNPs which were stabilized by oleic acid and immobilized amphiphilic zwitterionic polymers by hydrophobic interactions at the hydrophobic surface of the nanoparticles [133]. Other strategies which have been reported are to conduct emulsion polymerizations or the synthesis of MNP in the presence of polyzwitterions as shown by Mincheva et al. who simply added polyelectrolytes during the respective MNPs synthesis [134].

**Figure 5:**
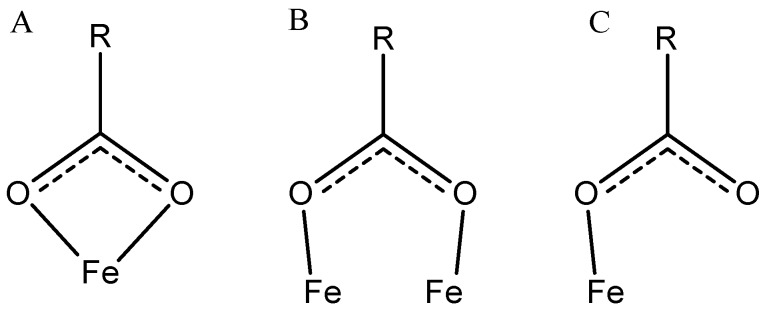
Carboxylate binding models: (**A**) bidentate chelate; (**B**) bidentate bridging; and (**C**) monodentate coordination. Reprinted from [137] with permission from ACS Publications.

There are two possible strategies for adsorptive surface modifications, either the adsorption of end-functionalized polyelectrolytes in analogy to the covalent grafting-to, or adsorption of the polyelectrolyte chain. The latter can be realized utilizing either the anionic groups of the polyelectrolyte itself, or special anchor groups which can be introduced by the formation of copolymers or block copolymers. Suitable anchor groups besides the functional groups present in the polyelectrolyte are for example catechol derivatives like dopamine [135], arsenic acid or phosphonates [136]. Among the groups which are used for immobilization one of the most prominent examples is the carboxyl group. Here, direct complexation of the iron oxide surface is possible in different ways (Figure 5). Usually, multiple carboxylic groups per polymer chain are used for the immobilization to deliberately avoid the detachment of the polymeric shell at low concentrations. The binding mode for each carboxylate can be bidentate chelate (A), bidentate bridging (B), or monodentate (C), and depends on the surrounding solution conditions (e.g., pH) as well as on the substituent (R) of the carboxylic acid [137].

**Figure 6:**
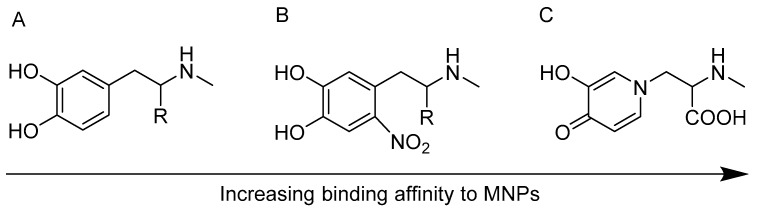
Catechol derivatives with increasing binding affinity to MNPs. (**A**) Catechol; (**B**) Nitrocatechol; (**C**) Mimosine. Reprinted from [140] with permission of ACS Publications.

Further prominent anchoring groups are catechols as this mimics the anchoring mechanism of marine mussels in nature, which use dopamin groups in their adhesive mussel foot proteins. A few examples use vinyl-catechols as one segment in block copolymers to facilitate anchoring at the surface of magnetic nanoparticles [138,139]. However, to our knowledge there are no examples so far for block copolymers consisting of a vinyl-catechol segment and a block of polyzwitterionic species. Instead there is an example where the catechol anchoring group appears only as an end group of polyzwitterions, as shown by Zhang et al. [135]. As catechols exhibit an extremely strong binding affinity to surfaces (especially to iron oxide) one catechol group per polymer allows in this case satisfying anchoring at the nanomaterial surface. Derivatives of catechol groups can also strongly influence the binding affinity to iron oxide surfaces. In general, catechol derivatives featuring electron withdrawing substituents lead to an enhanced binding affinity and thus to an enhanced stability of the resulting hybrid materials. Amstad et al. investigated different catechol-derived anchoring groups and were able to show that a stronger binding affinity does not necessarily result in an improved dispersion stability, but an optimal binding affinity of the anchors was identified (Figure 6). If the binding affinity is too strong, as in the example of applying mimosine as ligand system, the complexation can even lead to the removal of Fe^3+^-ions which gradually dissolves the nanoparticles [140].

Less frequently used anchoring groups for the immobilization of polymers at MNPs are phosphate anchoring groups. Miles et al. report in this context on the synthesis of MNPs which are modified by poly(ethylene glycol) (PEG) with different anchoring groups like monophosphonate and triphosphate and compared these to carboxylic acid moieties. The magnetite surface coverage was observed to be most satisfying in density and stability under physiological conditions with the triphosphate anchoring group. The observed grafting density is attributed to the three binding possibilities, resulting in an increased stabilization. Furthermore, phosphate groups show lower interactions with phosphate salts present under physiological conditions [141]. Similar observations concerning the anchoring stability of PEG-trisphosphate modified MNPs were made by Goff et al. [142]. Additional investigations of Maliakal et al. showed, that phosphonate groups form more stable bonds to metal oxide nanoparticles compared to carbonates [143].

The grafting method itself has a large impact on the properties of the resulting coating. Adsorption leads to the formation of thin monolayers, since further adsorption is inhibited due to the high surface concentration if compared to the surrounding solution, resulting in a rather high diffusion barrier [144,145]. Compared to that, polyelectrolytes which are bound to the NP surface with end-functionalities form thicker but typically less dense coatings. Nevertheless, typically the highest grafting densities can be achieved with grafting-from approaches [145].

## 5. Characterization Methods

**Figure 7:**
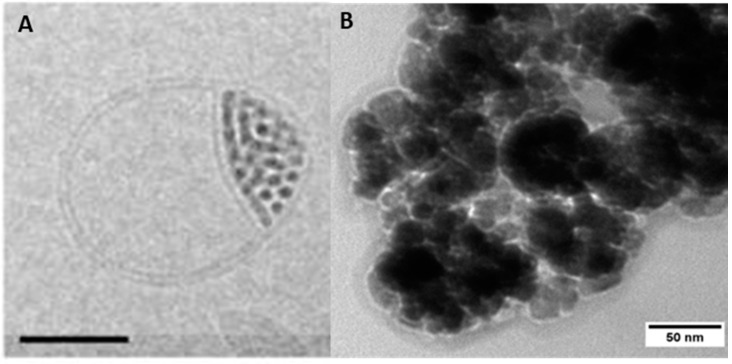
Cryo-TEM (transmission electron microscopy) micrographs of (**A**) lipid bilayer splitting around incorporated MNPs (scale bar = 50 nm). Reprinted from [151] with permission of ACS Publications. (**B**) Protein corona of bovine serum albumin formed at the surface of MNPs (unpublished own data).

**Figure 8:**
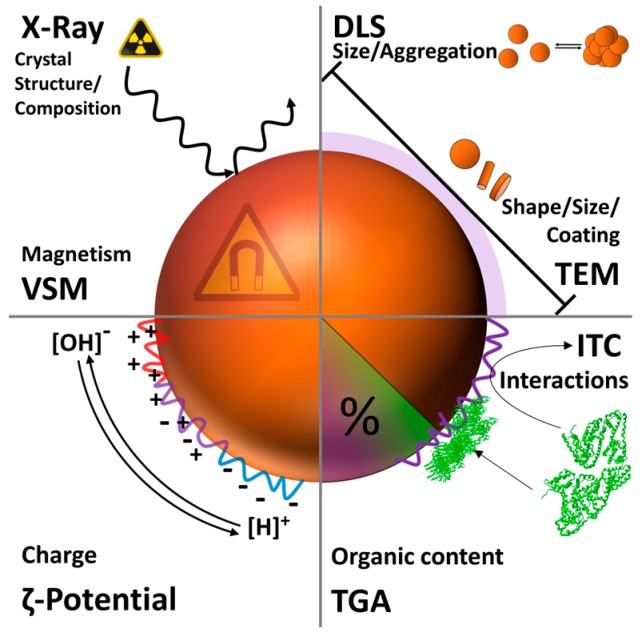
Schematic representation of different analysis techniques for magnetic nanoparticles and the corresponding surface modifications.

Several established methods exist for the investigation of nanoparticles or the corresponding hybrid materials. Herein we want to focus on characterization methods which mainly target shell thickness and shell characteristics as well as the altered properties of the core-shell construct after successful coating (Figure 7 and Figure 8).

As already mentioned in Section 2, both the core size and the size distribution have tremendous influence on the characteristics of any nanomaterial and, hence, reliable methods to determine these parameters are crucial. In that regard, dynamic light scattering (DLS) can be a useful tool. DLS uses Brownian motion to provide information about the hydrodynamic radius (*R_h_*), size distribution (polydispersity, PDI), and the colloidal stability of nanoparticles in solution [146]. Quite often, PDI values from 0.1 to 0.25 are used to confirm a narrow size distribution, whereas a PDI value higher than 0.5 is often referred to as a broad distribution [147]. The size distributions resulting from DLS are of high value concerning the aggregation behavior prior to and after surface modification as well as the apparent changes in nanoparticle size. However, this method merely provides an average value whereas transmission electron microscopy (TEM) provides supplementary information about size, shape, and shell thickness of individual nanoparticles or clusters thereof. Especially regarding the latter case, TEM investigations can be easily used to get an impression about the effect of the polymeric shell on the MNP aggregation behavior. However, the results have to be interpreted with care as aggregation of the nanoparticles and damaging of organic nanostructures can occur during drying processes. For this reason, TEM and DLS are often used in combination [148]. Additionally, cryo TEM has to be applied for samples which are sensitive to drying processes. Cryo TEM reveals structural information without drying the artifacts as the samples are measured in a vitrified aqueous surrounding. The aqueous sample is therefore vitrified by plunging into liquid ethane. This technique is of special interest when it comes to the visualization of clustering processes [149], samples which include liposome-like structures [150,151], or the visualization of biological interaction processes with the respective nanoparticles (Figure 7) [152].

The zeta potential of nanoparticles has tremendous influence on their suspension stability, eventual secondary aggregation, or any interaction with other materials. The zeta potential is measured by laser doppler velocimetry as the electrophoretic mobility of the respective colloidal suspension and represents the potential at the slipping plane of a particle in solution during movement [153]. In general, high values result in an improved stabilization, while a value close to zero typically leads to fast aggregation and eventual precipitation in aqueous media. Due to the adsorption of protein upon contact with biological media, the biological identity of nanoparticles can strongly differ from their synthetic identity concerning aggregation and surface charge [154]. Therefore, it is important to note that high zeta potential values are not necessarily an indication for dispersion stability in biological media. According to several reports, zwitterionic coatings seem to be beneficial with regard to dispersion stability over extended broad pH ranges and at different salt concentrations [4].

Powder X-ray investigations are most often used to obtain information about the crystal structure and phase of the magnetic core. This method provides information regarding the crystallinity of nanoparticles, as well as the average nanoparticle diameter. In addition, information on eventual crystalline organic shells can also be obtained but data analysis in these cases can be rather challenging [155]. The magnetic properties of magnetic nanoparticles are determined by vibrating sample magnetometry (VSM). The magnetic properties can be used to estimate the amount of diamagnetic material in the sample, for example the organic material representing the shell. Comparison of the weight of a sample with the corresponding magnetic properties allows calculation of the amount of diamagnetic organic material. Furthermore, this method validates whether the investigated nanoparticles are (still) superparamagnetic. Thermogravimetric analysis (TGA) can be used to determine the overall amount of organic material located at the surface of inorganic nanoparticles. Thereby, one clear benefit is that small samples amounts can be used to verify the presence of organic surface coatings. This tool is of utmost interest when it comes to a quantitative evaluation of coating processes and/or the determination of biological adsorption processes [156]. Isothermal titration calorimetry (ITC), usually a method used for the quantification of binding processes, is an attractive method to investigate interactions of MNPs with other molecules. This technique is often used for the quantification of interactions between small molecules and enzymes or DNA and has therefore the potential to quantify the adsorption of proteins or macromolecules onto the surface of nanoparticles. There are few examples in the literature of the investigation of magnetic nanoparticles using ITC but it can be used to determine the binding affinity *K_a_* (binding strength), the binding enthalpy Δ*H*, as well as the binding stoichiometry *n*. This allows, for example, for quantification of the protein repellence of a given nanomaterial [157,158,159,160].

## 6. Synthesis of Polyzwitterionic Shell Materials

The first polyampholytes were described in the 1950s by Alfrey, Fuoss, Morawetz, and Pinner as copolymers of methacrylic acid and either 2/4-vinyl pyridine or *N*,*N*-diethylamino methacrylate [161,162]. The first synthetic polyzwitterion matching the previously mentioned IUPAC definition was described by Harry Ladenheim and Herbert Morawetz, who synthesized poly(4-vinyl pyridine betaine) by quaternization of poly(4-vinyl pyridine) with ethylbromoacetate and subsequent hydrolysis of the resulting ester in 1957 [163]. After these first approaches, a lot of progress was made in the synthesis of polyzwitterions by various techniques. Most noticeable in our opinion is the utilization of controlled polymerization techniques and the large variety of monomers which has been made accessible. Controlled polymerization techniques not only allow control over molar mass, dispersity, and polymer architecture but also provide access to block copolymers featuring polyzwitterionic or polyampholytic blocks [164,165,166,167]. For a detailed overview on synthetic access and properties of polyzwitterions we refer the reader to an excellent recent review article [101]. However, many approaches today using polyzwitterions as coating materials for MNPs still report on polymer-analogous reactions like for example the quaternization of poly(4-vinyl pyridine) to generate zwitterionic polymers [132,134,136,164,168,169]. Quite often the dispersity of the polymers used for surface functionalization is of secondary importance. This can be of advantage if polymerization is impeded by certain groups which have to be protected prior to polymerization or when polymers are of interest but naturally not of polyzwitterionic character. Commonly used techniques in that respect are different protection/deprotection strategies for different functional groups, quaternization of amines (often coupled with the introduction of anionic moieties, resulting in the formation of betaines), or esterification as an intermediate step. Some polyzwitterions can also be obtained by direct polymerization of the corresponding monomer without any subsequent modification being necessary [135,166]. In most cases, nanoparticle synthesis and surface functionalization are two separate steps which have the advantage that the properties and characteristics of the respective building blocks can be adjusted (and investigated) separately prior to the formation of core-shell hybrid materials. On the other hand, direct one-pot approaches can reduce the overall synthetic efforts and are attractive concerning scalability. In the following, polyzwitterions and, in one case, a polyampholyte which were used for coating of magnetic nanoparticles are discussed. They are listed and arranged according to the techniques used for immobilization on the MNP surface.

### 6.1. Covalent Surface Functionalization

In the first section, covalently grafted polyzwitterions are discussed. The examples are summarized in Table 1.

**Figure 9:**
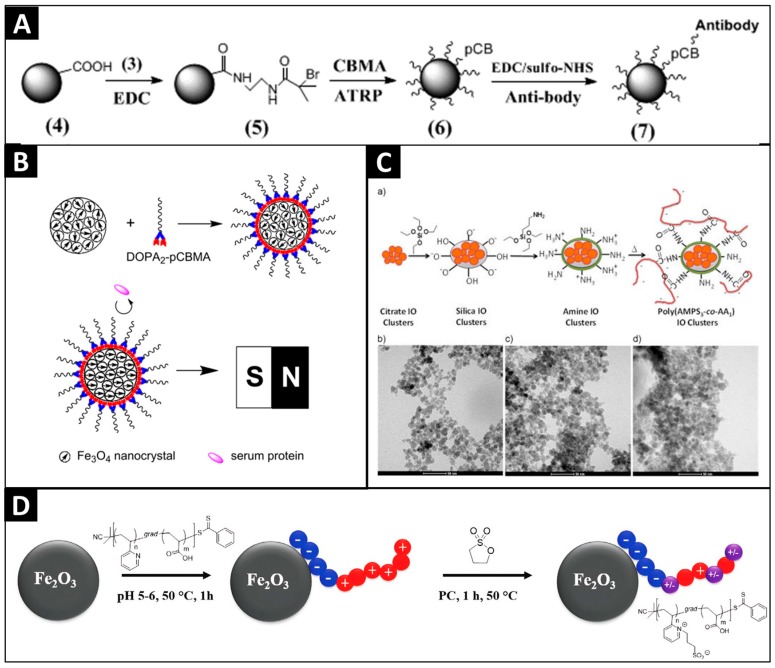
(**A**) Grafting-from approach for the polymerization of CBMA (carboxybetaine methacrylate). Reprinted from [130] with permission of ACS Publications; (**B**) Preparation of pCBMA-DOPA-2-MNPs and their magnetization in the presence of a permanent magnet. Reprinted from [135] with permission of Elsevier; (**C**) Scheme of the synthesis of poly(AMPS-*co*-AA) MNPs. Reprinted from [128] with permissions of ACS Publications; (**D**) Scheme of grafting-to of P(2VP-*grad*-AA) onto MNP and subsequent sultonation of P(2VP-*grad*-AA)@MNP, reprinted from [164] with permission of John Wiley and Sons.

Urena-Benavides et al. formed iron oxide nanoclusters with silica shells, which were then functionalized with amino groups on the surface using 3-aminopropyl triethoxysilane (**1**). The amino groups were used to covalently graft a poly(2-acrylamido-3-methylpropanesulfonate-*co*-acrylic acid) copolymer to the nanoparticle surface. The resulting hybrid particles showed reduced adsorption to porous materials (Figure 9C) [128]. Zhang et al. prepared an ATRP initiator bearing an amine functionality at the chain end, which was used for surface immobilization of the initiator onto superparamagnetic nanospheres. The initiator was then used for the surface initiated polymerization of carboxybetaine methacrylate (CBMA **2**). Furthermore, both pristine and PCBMA functionalized MNP were further functionalized with antibodies of the β subunit of human chorionic gonadotropin (anti-β-hCG). The particles showed reduced non-specific protein adsorption, and have high potential for biosensing applications (Figure 9A) [130]. An example of grafting-through surface functionalization was presented by Chen et al. (**3**). They published the synthesis of polyzwitterion coated magnetic nanoparticles via a grafting-through approach. At first, the magnetite nanoparticles were coated with a thin silica shell using the Stöber-process, followed by grafting with 3-(trimethoxysilyl)propyl methacrylate (γ-MPS), creating reactive double-bonds on the nanoparticle surface. The zwitterionic shell was then synthesized by copolymerization of methacrylic acid (MAA), *N,N'*-methylenebisacrylamide (MBA) as crosslinker, and 2-(methacryloyloxy)ethyl-dimethyl-(3-sulfopropyl) ammonium hydroxide (MSA) as zwitterionic co-monomer [131].

### 6.2. Electrostatic Adsorption

In the following section, polyzwitterions, which were adsorbed onto MNP will be discussed, and the shown examples are summarized in Table 2.

Although we mainly focus on polyzwitterionic coating materials, here we also included one example of a polyampholyte to show that the resulting hybrid materials can show similar properties to the examples discussed before. Xiao et al. coated in a first step iron oxide nanoparticles with poly(acrylic acid) (PAA) and modified them in a second step by esterification with 3-(diethylamino)propylamine, resulting in a polyampholytic shell material. The resulting nanoparticles exhibited low macrophage cell uptake and low cell toxicity (**4**) [170]. Billing et al. showed one of the few examples where controlled polymerization techniques were applied to generate polyzwitterions as coating materials for MNPs. Using reversible addition–fragmentation chain transfer (RAFT)-polymerization, gradient copolymers consisting of 2-vinyl pyridine and *tert*-butyl acrylate (poly(2-vinylpyridine-grad-*tert*-butylacrylate)) were prepared (**5**). Subsequently, the *tert*-butylgroups were hydrolyzed to acrylic acid and the 2-vinylpyridine moieties were sultonated to generate a zwitterionic unit (Figure 9D). As a result of the functionalization, an increased stability towards secondary aggregation was observed and cytotoxicity tests did not show a significant influence on cell viability [164]. Von der Lühe et al. showed the synthesis of zwitterionic polydehydroalanine (**6**). This polymer exhibits a high charge to volume ratio as it consists of a polymeric backbone with directly attached amine and carboxylic acid functionalities. As these functional groups would impede direct polymerization, both functionalities had to be protected prior to polymerization. The protective groups were cleaved off afterwards to generate a polyzwitterion and the carboxyl groups were used for immobilization at the surface of sub 10 nm MNPs [132] and multicore nanoparticles with 80 nm in diameter. The PDha@MC particles were further used for the adsorption and selective desorption of both polyanions and polycations [171]. Zhu et al. used O-carboxymethylchitosan as a naturally occurring polysaccharide and modified the material by functionalization with carboxylic acid groups, followed by immobilization at the surface of MNPs. The resulting nanoparticles were well dispersed in aqueous media and showed good cytocompatibility (**7**) [168]. Besides carboxylic acids, other functionalities like catechols, phosphonates, or oligoglycols can be used for the immobilization of polyzwitterions on nanoparticle surfaces. Dopamine was used by Zhang et al. who synthesized a double-dopamine functionalized ATRP initiator, where all catecholic moieties were protected with *tert*-butyldimethylsilyl ethers (TBDMS, Figure 9B) (**8**). The initiator was then used for the polymerization of carboxybetainemethacrylate (CBMA). After deprotection of the catecholic hydroxyl groups, the polyzwitterion was used to coat iron oxide MNPs. The resulting hybrids showed increased dispersion stability in solutions of varying ionic strength and blood serum compared to pristine and citrate stabilized MNPs. Furthermore, macrophage uptake was drastically decreased [135]. Yuan et al. synthesized poly[2-(methacryloyloxy)ethyl phosphorylcholine]-*block*-(glycerol monomethacrylate) (PMPC-*b*-PGMA) block copolymers by ATRP (**9**). The double-hydrophilic block copolymer was added to a co-precipitation of FeCl_2_ and FeCl_3_.The bis-hydroxides of the PGMA block ensured efficient immobilization of the polymer on the surface of the resulting nanoparticles, and the zwitterionic block increased long term-stability [166].

### 6.3. Other Methods

In this last section, less frequently employed functionalization methods, like pre-functionalization approaches with polyelectrolytes, utilizing hydrophilic/hydrophobic interactions, or the addition of polyzwitterions during MNP preparation are discussed. The discussed examples are summarized in Table 3.

The use of non-covalent interactions (electrostatic or hydrophobic-hydrophilic interactions) to immobilize polyelectrolytes at the surface of MNPs leads to systems which allow the detachment of the respective polymeric shell under specific conditions, which can be either a benefit or a drawback. In order to generate a high surface charge at the surface of MNPs, Yeh et al. used poly(acrylic acid) as a first layer. By applying poly(4-vinylpyridinium *N*-ethylsulfonate), attractive electrostatic interactions led to the formation of a second layer (**10**). It is noteworthy that the direct attachment of the polyzwitterion is also possible without the underlying PAA layer but the resulting surface coating was by far less stable afterwards [172]. V. G. Demillo et al. took advantage of hydrophilic hydrophobic interactions. They produced multifunctional magnetofluorescent NPs by encapsulating quantum dots and MNPs within a polymeric shell. Poly(maleic anhydride-*alt*-1-octadecene) (PMAO) was used as precursor and modified by opening the anhydrous rings in the polymer in a first step with 3-(dimethyl-amino)-1-propylamine (**11**). In a second step the generated tertiary amines were reacted with β-propiolactone and 1,3-propanesultone resulting in betaine structures. As the polymer backbone has an amphiphilic character these polymers were immobilized at the nanoparticles by using hydrophilic hydrophobic interactions between the polyampholytes and the hydrophobic nanoparticles [169]. A similar approach was performed by Wang et al. who prepared microspheres of chitosan and poly(aspartic acid) with encapsulated magnetic nanoparticles and CdTe quantum dots (**12**). The 110–320 nm large microspheres are of interest in the context of biolabeling and imaging [173]. Pombo-Garcia et al. utilized hydrophobic interactions for the functionalization of ultra small superparamagnetic iron oxide nanoparticles with poly(maleic anhydride-*alt*-1-decene), which was previously substituted with 3-(dimethylamino)propylamine to give a zwitterionic polymer (PMAL) (**13**). The surface coating was realized by intercalation of decene with previously attached oleic acid [133]. The resulting hybrids were characterized concerning protein adsorption and biocompatibility. R. Mincheva et al. showed the in-situ formation of polyzwitterion-coated magnetic nanoparticles by adding the polymeric shell material during the synthesis of MNPs. The two biocompatible polyelectrolytes (*N*-carboxyethylchitosan (CECh) (**14**) and poly(2-acrylamido-2-methylpropanesulfonic acid) (PAMPS) (**15**) are capable of stabilizing MNPs in aqueous solution. CECh was synthesized by a polymer-analogous reaction with acrylic acid, while PAMPS was synthesized directly by free radical polymerization of 2-acrylamido-2-methylpropanesulfonic acid. Here, both suspension stability and particle size as well as the resulting magnetic properties were investigated and the obtained nanocomposites were further used for electrospinning [134].

## 7. A Short Note on Application Fields

Different applications of specific core-shell combinations have been already showcased throughout the last chapters. Nevertheless, by far the highest application potential for polyzwitterion-coated magnetic nanoparticles in our opinion is within the field of biomedical applications. As demonstrated in Section 2, magnetic cores are of high interest for applications like MRI imaging, drug delivery, and hyperthermia [170]. This potential might even be increased with polyzwitterionic coatings, since the circulation times can be prolonged and secondary (unspecific) aggregation is prevented.

Further, these materials (especially the multicore iron oxide NPs) are promising with regard to bioseparation approaches as the magnetic nanoparticles enable a facile and fast way of binding and separating biomolecules (e.g., glycopeptides) from complex biological systems by external magnetic fields. As this allows an enrichment of the respective molecules, rather high detection sensitivities can be achieved. Further analysis of any separated molecules or macromolecules can afterwards be realized by techniques such as mass spectrometry or various spectroscopy methods [131,172,174].

Besides biomedical applications, the polyzwitterionic magnetic hybrid materials are also constantly discussed with regard to technical applications such as extraction processes (e.g., wastewater treatment or organic pollutant extraction) [136], as the zwitterionic surface enables adsorption of cationic metal ions, which could possibly be released by changes in pH. The benefit of the magnetic cores in this case is again the possibility of mechanical manipulation, in particular the separation from dispersions by an external magnetic field. This property also renders these materials interesting for catalytic processes, as such heterogeneous catalysts can be easily separated, purified if necessary, and reused in further cycles [175]. Finally, magnetic imaging is also of interest in other fields like subsurface imaging. Here, the low tendency for interaction with surrounding materials of different polarity enables the use of polyzwitterionic surface coatings on MNPs in imaging for oil recovery as shown by Ureña-Benavides et al. [128].

## 8. Conclusions and Outlook

The synthesis and exploitation of magnetic hybrid materials–in our case consisting of a magnetic core and an organic shell—has already arrived in a broad variety of research areas. However, still only a certain number of research groups have reported on the use of polyzwitterions as coating materials, which we mainly attribute to the fact that the synthesis of polyzwitterions can be challenging and that PEG still is the most prominent biocompatible shell material in many applications. Nevertheless, magnetic hybrid materials which are functionalized by polyzwitterions show several benefits compared to the gold standard PEG, like close similarities to biological tissue, multiple ways of immobilization, and, in some cases, pH responsive behavior rendering those examples interesting candidates for drug delivery systems in the near future. The adjustment of charge at the particle surface allows a potential change in solubility of the particles as well as a change in adsorptive behavior towards any suitable guest molecules (or cargo).

Further advance in the context of biomedical applications clearly requires progress concerning the understanding of interactions with proteins and biological macromolecules. Along the same line, a closer look at the influence of the actual combination of strong and weak polyelectrolyte building blocks on the resulting interactions with biological tissues has to be taken as well. The qualitative as well as the quantitative binding of different proteins to the surface of the respective materials might give further information on processes which are important in understanding the governing factors in protein corona formation. Furthermore, basic investigations on the suspension stability depending on ionic strength, and the response to the presence of different counter ions or biological fluids are further important aspects. The examples outlined above also suggest that combinations of PEG and polyzwitterions within polymeric shells are definitely an aspect of interest.

## Figures and Tables

**Table 1:** Structures, binding method, potential application (if provided) and type of polyelectrolyte combination for polyzwitterions which were used for covalent surface functionalization of magnetic nanoparticles (MNP).

Nr.	Polyzwitterionic Unit Structure/Name	Binding Method	Application	Type of Polyelectrolyte +/−
**1**	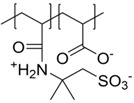 P(AMPS-*co*-AA) [128]	covalent attachment via grafting-to to amino-silylated particles		weak/strong
**2**	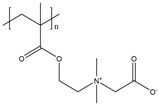 PCBMA [130]	grafting-from	Sensing	Strong/weak
**3**	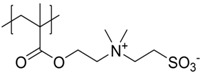 PMSA [131]	grafting-through using γ-MPS (silylation)	Isolation of glycoptides from biological samples (bioseparation)	strong/strong

**Table 2:** Structures, binding method, potential application (if provided) and type of polyelectrolyte combination for polyzwitterions which were used for adsorptive surface functionalization of MNP.

Nr.	Polyzwitterionic Unit Structure/Name	Binding Method	Application	Type of Polyelectrolyte +/−
**4**	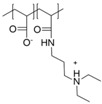 P(AA-*stat*-PDEAPA) [170]	carboxyl group anchoring, grafting-to		weak/weak
**5**	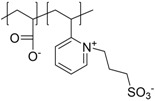 P(2VP-*grad*-AA) [164]	carboxyl group anchoring, grafting-to	antifouling	weak/weak
**6**	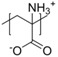 PDha[132]	carboxyl group anchoring, grafting-to	antifouling	weak/weak
**7**	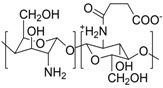 NSOCMS [168]	carboxyl group anchoring, grafting-to		weak/weak
**8**	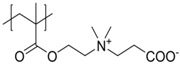 PCBMA[135]	Catechol anchoring, grafting-to		strong/weak
**9**	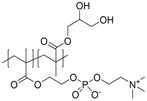 PMPC-*b*-PGMA [166]	adsorption via bishydroxides of the PGMA block, grafting-to		strong/weak

**Table 3:** Structures, binding method, potential application (if provided), and type of polyelectrolyte combination for polyzwitterions which were used for surface functionalization of MNP via the formation of polyelectrolyte complexes, hydrophilic/hydrophobic interactions, or by addition as surfactant during co precipitation.

Nr.	Polyzwitterionic Unit Structure/Name	Binding Method	Application	Type of Polyelectrolyte +/−
**10**	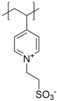 P4VP-SB [172]	electrostatic interactions PAA@MNP	isolation of glycopeptides from biological samples (bio-separation)	strong/strong
**11**	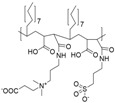 PMAO-CB-SB [169]	hydrophilic hydrophobic interactions	cancer diagnosis	strong/weak
**12**	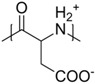 PAsp [173]	polyelectrolyte complexes between chitosan (CS) and poly(aspartic acid) (PAsp) with encapsulated MNP		weak/weak
**13**	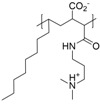 PMAL [133]	hydrophilic hydrophobic interactions	anti-fouling	weak/weak
**14**	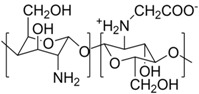 CeCh [134]	adsorption via carboxylates	electro spinning	weak/weak
**15**	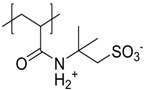 PAMPS [134]	sulfonate anchoring	electro spinning	weak/strong

## References

[1] Veiseh O., Gunn J.W., Zhang M. (2010). Design and fabrication of magnetic nanoparticles for targeted drug delivery and imaging. Adv. Drug Deliv. Rev..

[2] Cao B., Tang Q., Cheng G. (2014). Recent advances of zwitterionic carboxybetaine materials and their derivatives. J. Biomater. Sci..

[3] Wu W., Wu Z., Yu T., Jiang C., Kim W.-S. (2015). Recent progress on magnetic iron oxide nanoparticles: Synthesis, surface functional strategies and biomedical applications. Sci. Technol. Adv. Mater..

[4] García K.P., Zarschler K., Barbaro L., Barreto J.A., O’Malley W., Spiccia L., Stephan H., Graham B. (2014). Zwitterionic-Coated “Stealth” Nanoparticles for Biomedical Applications: Recent Advances in Countering Biomolecular Corona Formation and Uptake by the Mononuclear Phagocyte System. Small.

[5] Liu T.-Y., Hu S.-H., Liu D.-M., Chen S.-Y., Chen I.W. (2009). Biomedical nanoparticle carriers with combined thermal and magnetic responses. Nano Today.

[6] Ai H. (2011). Layer-by-layer capsules for magnetic resonance imaging and drug delivery. Adv. Drug Deliv. Rev..

[7] Xing R., Liu G., Zhu J., Hou Y., Chen X. (2014). Functional Magnetic Nanoparticles for Non-Viral Gene Delivery and MR Imaging. Pharm. Res..

[8] Bodker F., Morup S., Linderoth S. (1994). Surface effects in metallic iron nanoparticles. Phys. Rev. Lett..

[9] Hacliipanayis C.G., Bonder M.J., Balakrishanan S., Wang X., Mao H., Hadjipanayis G.C. (2008). Metallic Iron Nanoparticles for MRI Contrast Enhancement and Local Hyperthermia. Small.

[10] Li Y.F., Hu Y.J., Huang G.J., Li C.Z. (2013). Metallic iron nanoparticles: Flame synthesis, characterization and magnetic properties. Particuology.

[11] Yamamuro S., Ando T., Sumiyama K., Uchida T., Kojima I. (2004). Monodisperse Metallic Iron Nanoparticles Synthesized from Noncarbonyl Complex. Jpn. J. Appl. Phys. Part 1.

[12] Seong G., Takami S., Arita T., Minami K., Hojo D., Yavari A.R., Adschiri T. (2011). Supercritical hydrothermal synthesis of metallic cobalt nanoparticles and its thermodynamic analysis. J. Supercrit. Fluids.

[13] Zeisberger M., Dutz S., Müller R., Hergt R., Matoussevitch N., Bönnemann H. (2007). Metallic cobalt nanoparticles for heating applications. J. Magn Magn. Mater..

[14] Tran N., Webster T.J. (2010). Magnetic nanoparticles: Biomedical applications and challenges. J. Mater. Chem..

[15] Lee J.G., Li P., Dong X.L., Choi C.J. (2010). Fabrication of Ferromagnetic Mn-Al Alloy Nanoparticles Using a Plasma Arc-discharge Process. Korean J. Met. Mater..

[16] Wang C.H., Meyer J., Teichert N., Auge A., Rausch E., Balke B., Hutten A., Fecher G.H., Felser C. (2014). Heusler Nanoparticles for Spintronics and Ferromagnetic Shape Memory Alloys. J. Vac. Sci. Technol. B.

[17] White C.W., Withrow S.P., Sorge K.D., Meldrum A., Budai J.D., Thompson J.R., Boatner L.A. (2003). Oriented Ferromagnetic Fe-Pt Alloy Nanoparticles Produced in Al_2_O_3_ by Ion-Beam Synthesis. J. Appl. Phys..

[18] Lasheras X., Insausti M., de Muro I.G., Garaio E., Plazaola F., Moros M., De Matteis L., de la Fuente J.M., Lezama L. (2016). Chemical Synthesis and Magnetic Properties of Monodisperse Nickel Ferrite Nanoparticles for Biomedical Applications. J. Phys. Chem. C.

[19] Rodrigues A.R.O., Gomes I.T., Almeida B.G., Araujo J.P., Castanheira E.M.S., Coutinho P.J.G. (2015). Magnetic liposomes based on nickel ferrite nanoparticles for biomedical applications. Phys. Chem. Chem. Phys..

[20] Ruthradevi T., Akbar J., Kumar G.S., Thamizhavel A., Kumar G.A., Vatsa R.K., Dannangoda G.C., Martirosyan K.S., Girija E.K. (2017). Investigations on nickel ferrite embedded calcium phosphate nanoparticles for biomedical applications. J. Alloy. Compd..

[21] Tomitaka A., Jeun M., Bae S., Takemura Y. (2011). Evaluation of Magnetic and Thermal Properties of Ferrite Nanoparticles for Biomedical Applications. J. Magn..

[22] Peeples B., Goornavar V., Peeples C., Spence D., Parker V., Bell C., Biswal D., Ramesh G.T., Pradhan A.K. (2014). Structural, Stability, Magnetic, and Toxicity Studies of Nanocrystalline Iron Oxide and Cobalt Ferrites for Biomedical Applications. J. Nanopart. Res..

[23] Salunkhe A.B., Khot V.M., Thorat N.D., Phadatare M.R., Sathish C.I., Dhawale D.S., Pawar S.H. (2013). Polyvinyl alcohol functionalized cobalt ferrite nanoparticles for biomedical applications. Appl. Surf. Sci..

[24] Sanpo N., Berndt C.C., Wen C., Wang J. (2013). Transition metal-substituted cobalt ferrite nanoparticles for biomedical applications. Acta Biomater..

[25] Cornell R.M., Schwertmann U. (2003). The Iron Oxides: Structure, Properties, Reactions, Occurences and Uses.

[26] Khalafalla S.E., Reimers G.W. (1980). Preparation of Dilution-Stable Aqueous Magnetic Fluids. IEEE Trans. Magn..

[27] Massart R. (1980). Preparation of aqueous ferrofluids without using surfactant—Behavior as a function of the pH and the counterions. C. R. Seances Acad. Sci. Ser. C.

[28] Krishnan K.M. (2010). Biomedical Nanomagnetics: A Spin through Possibilities in Imaging, Diagnostics, and Therapy. IEEE Trans. Magn..

[29] Pankhurst Q.A., Thanh N.T.K., Jones S.K., Dobson J. (2009). Progress in Applications of Magnetic Nanoparticles in Biomedicine. J. Phys. D.

[30] Bloch F. (1932). Zur Theorie des Austauschproblems und der Remanenzerscheinung der Ferromagnetika. Z. Phys..

[31] Weiss P. (1907). L’hypothese du Champ Moleculaire et la Propriete Ferromagnetique. J. Phys. Radium.

[32] Landau L.D., Lifshitz E.M. (1935). On the Theory of the Dispersion of Magnetic Permeability in Ferromagnetic Bodies. Phys. Z. Sowjetunion.

[33] Kittel C. (1946). Theory of the Structure of Ferromagnetic Domains in Films and Small Particles. Phys. Rev..

[34] Butler R.F., Banerjee S.K. (1975). Theoretical Single-Domain Grain Size Range in Magnetite and Titanomagnetite. J. Geophys. Res..

[35] Fabian K., Kirchner A., Williams W., Heider F., Leibl T., Hubert A. (1996). Three-dimensional micromagnetic calculations for magnetite using FFT. Geophys. J. Int..

[36] Dutz S. (2008). Nanopartikel in der Medizin.

[37] Néel L. (1949). Influence des fluctuations thermiques a l’aimantation des particules ferromagnetiques. C. R. Acad. Sci..

[38] Dutz S. (2016). Are Magnetic Multicore Nanoparticles Promising Candidates for Biomedical Applications?. IEEE Trans. Magn..

[39] Dutz S., Andrä W., Hergt R., Müller R., Oestreich C., Schmidt C., Töpfer J., Zeisberger M., Bellemann M.E. (2007). Influence of Dextran Coating on the Magnetic Behaviour of Iron Oxide Nanoparticles. J. Magn. Magn. Mater..

[40] Suzdalev I.P., Maksimov Y.V., Buravtsev V.N., Imshennik V.K., Kazakevich A.G., Novichikhin S.V. (2000). The formation and properties of a system of iron oxide nanoclusters. Colloid J..

[41] Blanco-Andujar C., Ortega D., Southern P., Pankhurst Q.A., Thanh N.T.K. (2015). High Performance Multi-core Iron Oxide Nanoparticles for Magnetic Hyperthermia: Microwave Synthesis, and the Role of Core-to-Core Interactions. Nanoscale.

[42] Dutz S., Kettering M., Hilger I., Müller R., Zeisberger M. (2011). Magnetic multicore nanoparticles for hyperthermia-influence of particle immobilization in tumour tissue on magnetic properties. Nanotechnology.

[43] Lartigue L., Hugounenq P., Alloyeau D., Clarke S.P., Levy M., Bacri J.-C., Bazzi R., Brougham D.F., Wilhelm C., Gazeau F. (2012). Cooperative Organization in Iron Oxide Multi-Core Nanoparticles Potentiates Their Efficiency as Heating Mediators and MRI Contrast Agents. ACS Nano.

[44] Hergt R., Dutz S., Roder M. (2008). Effects of size distribution on hysteresis losses of magnetic nanoparticles for hyperthermia. J. Phys. Condens. Matter.

[45] Müller R., Dutz S., Neeb A., Cato A.C.B., Zeisberger M. (2013). Magnetic heating effect of nanoparticles with different sizes and size distributions. J. Magn. Magn. Mater..

[46] Barry S.E. (2008). Challenges in the Development of Magnetic Particles for Therapeutic Applications. Int. J. Hyperth..

[47] Laurent S., Forge D., Port M., Roch A., Robic C., Elst L.V., Müller R.N. (2008). Magnetic Iron Oxide Nanoparticles: Synthesis, Stabilization, Vectorization, Physicochemical Characterizations, and Biological Applications. Chem. Rev..

[48] Lu A.H., Salabas E.L., Schüth F. (2007). Magnetic Nanoparticles: Synthesis, Protection, Functionalization, and Application. Angew. Chem. Int. Ed..

[49] Reddy L.H., Arias J.L., Nicolas J., Couvreur P. (2012). Magnetic Nanoparticles: Design and Characterization, Toxicity and Biocompatibility, Pharmaceutical and Biomedical Applications. Chem. Rev..

[50] Krishnan K.M. (2016). Fundamentals and Applications of Magnetic Materials.

[51] Bazylinski D.A., Garrattreed A.J., Frankel R.B. (1994). Electron microscopic studies of magnetosomes in magnetotactic bacteria. Microsc. Res. Tech..

[52] Faivre D., Schuler D. (2008). Magnetotactic Bacteria and Magnetosomes. Chem. Rev..

[53] Schüler D., Frankel R.B. (1999). Bacterial magnetosomes: Microbiology, biomineralization and biotechnological applications. Appl. Microbiol. Biotechnol..

[54] Timko M., Molcan M., Hashim A., Skumiel A., Müller M., Gojzewski H., Jozefczak A., Kovac J., Rajnak M., Makowski M. (2013). Hyperthermic Effect in Suspension of Magnetosomes Prepared by Various Methods. IEEE Trans. Magn..

[55] Hergt R., Hiergeist R., Zeisberger M., Schuler D., Heyen U., Hilger I., Kaiser W.A. (2005). Magnetic Properties of Bacterial Magnetosomes as Potential Diagnostic and Therapeutic Tools. J. Magn. Magn. Mater..

[56] Molcan M., Gojzewski H., Skumiel A., Dutz S., Kovac J., Kubovcikova M., Kopcansky P., Vekas L., Timko M. (2016). Energy Losses in Mechanically Modified Bacterial Magnetosomes. J. Phys. D.

[57] Baumgartner J., Carillo M.A., Eckes K.M., Werner P., Faivre D. (2014). Biomimetic Magnetite Formation: From Biocombinatorial Approaches to Mineralization Effects. Langmuir.

[58] Scheffel A., Gruska M., Faivre D., Linaroudis A., Plitzko J.M., Schüler D. (2006). An acidic protein aligns magnetosomes along a filamentous structure in magnetotactic bacteria. Nature.

[59] DeCastro C.L., Mitchell B.S., Baraton M.-I. (2002). Nanoparticles from Mechanical Attrition. Synthesis, Functionalization, and Surface Treatment of Nanoparticles.

[60] Dutz S., Hergt R., Mürbe J., Müller R., Zeisberger M., Andrä W., Töpfer J., Bellemann M.E. (2007). Hysteresis Losses of Magnetic Nanoparticle Powders in the Single Domain Size Range. J. Magn. Magn. Mater..

[61] Kurland H.-D., Grabow J., Staupendahl G., Andrä W., Dutz S., Bellemann M.E. (2007). Magnetic Iron Oxide Nanopowders Produced by CO_2_ Laser Evaporation. J. Magn. Magn. Mater..

[62] Kurland H.-D., Grabow J., Staupendahl G., Müller F.A., Müller E., Dutz S., Bellemann M.E. (2009). Magnetic iron oxide nanopowders produced by CO_2_ laser evaporation—‘In situ’ coating and particle embedding in a ceramic matrix. J. Magn. Magn. Mater..

[63] Stötzel C., Kurland H.-D., Grabow J., Dutz S., Müller E., Sierka M., Müller F.A. (2013). Control of the Crystal Phase Composition of Fe_x_O_y_ Nanopowders Prepared by CO_2_ Laser Vaporization. Cryst. Growth Des..

[64] Grüttner C., Müller K., Teller J., Westphal F., Foreman A., Ivkov R. (2007). Synthesis and Antibody Conjugation of Magnetic Nanoparticles with Improved Specific Power Absorption Rates for Alternating Magnetic Field Cancer Therapy. J. Magn. Magn. Mater..

[65] Dutz S., Clement J.H., Eberbeck D., Gelbrich T., Hergt R., Müller R., Wotschadlo J., Zeisberger M. (2009). Ferrofluids of magnetic multicore nanoparticles for biomedical applications. J. Magn. Magn. Mater..

[66] Sun S., Zeng H. (2002). Size-Controlled Synthesis of Magnetite Nanoparticles. J. Am. Chem. Soc..

[67] Kuckelhaus S., Garcia V.A.P., Lacava L.M., Azevedo R.B., Lacava Z.G.M., Lima E.C.D., Figueiredo F., Tedesco A.C., Morais P.C. (2003). Biological investigation of a citrate-coated cobalt-ferrite-based magnetic fluid. J. Appl. Phys..

[68] Hyeon T., Lee S.S., Park J., Chung Y., Bin Na H. (2001). Synthesis of highly crystalline and monodisperse maghemite nanocrystallites without a size-selection process. J. Am. Chem. Soc..

[69] Park J., Lee E., Hwang N.M., Kang M.S., Kim S.C., Hwang Y., Park J.G., Noh H.J., Kini J.Y., Park J.H. (2005). One-nanometer-scale size-controlled synthesis of monodisperse magnetic iron oxide nanoparticles. Angew. Chem. Int. Ed..

[70] Zhou Z.H., Wang J., Liu X., Chan H.S.O. (2001). Synthesis of Fe_3_O_4_ nanoparticles from emulsions. J. Mater. Chem..

[71] Okoli C., Sanchez-Dominguez M., Boutonnet M., Jaras S., Civera C., Solans C., Kuttuva G.R. (2012). Comparison and Functionalization Study of Microemulsion-Prepared Magnetic Iron Oxide Nanoparticles. Langmuir.

[72] Zeng Q., Baker I., Loudis J.A., Liao Y., Hoopes P.J., Weaver J.B. (2007). Fe/Fe oxide nanocomposite particles with large specific absorption rate for hyperthermia. Appl. Phys. Lett..

[73] Hao Y.L., Teja A.S. (2003). Continuous hydrothermal crystallization of α-Fe_2_O_3_ and Co_3_O_4_ nanoparticles. J. Mater. Res..

[74] Lv Y.D., Wang H., Wang X.F., Bai J.B. (2009). Synthesis, characterization and growing mechanism of monodisperse Fe_3_O_4_ microspheres. J. Cryst. Growth.

[75] Chen D., Xu R. (1998). Hydrothermal synthesis and characterization of nanocrystalline Fe_3_O_4_ powders. Mater. Res. Bull..

[76] Xu C., Teja A.S. (2008). Continuous hydrothermal synthesis of iron oxide and PVA-protected iron oxide nanoparticles. J. Supercrit. Fluids.

[77] Hugounenq P., Levy M., Alloyeau D., Lartigue L., Dubois E., Cabuil V., Ricolleau C., Roux S., Wilhelm C., Gazeau F. (2012). Iron Oxide Monocrystalline Nanoflowers for Highly Efficient Magnetic Hyperthermia. J. Phys. Chem. C.

[78] Müller R., Hergt R., Dutz S., Zeisberger M., Gawalek W. (2006). Nanocrystalline Iron Oxide and Ba Ferrite Particles in the Superparamagnetism-Ferromagnetism Transition Range with Ferrofluid Applications. J. Phys. Condens. Matter.

[79] Faraji M., Yamini Y., Rezaee M. (2010). Magnetic Nanoparticles: Synthesis, Stabilization, Functionalization, Characterization, and Applications. J. Iran. Chem. Soc..

[80] Xu J., Yang H.B., Fu W.Y., Du K., Sui Y.M., Chen J.J., Zeng Y., Li M.H., Zou G. (2007). Preparation and Magnetic Properties of Magnetite Nanoparticles by Sol-Gel Method. J. Magn. Magn. Mater..

[81] Alexiou C., Arnold W., Klein R.J., Parak F.G., Hulin P., Bergemann C., Erhardt W., Wagenpfeil S., Lubbe A.S. (2000). Locoregional Cancer Treatment with Magnetic Drug Targeting. Cancer Res..

[82] Lubbe A.S., Bergemann C., Riess H., Schriever F., Reichardt P., Possinger K., Matthias M., Dorken B., Herrmann F., Gurtler R. (1996). Clinical Experiences with Magnetic Drag Targeting: A Phase I Study With 4′-Epidoxorubicin in 14 Patients with Advanced Solid Tumors. Cancer Res..

[83] Gleich B., Weizenecker R. (2005). Tomographic Imaging Using the Nonlinear Response of Magnetic Particles. Nature.

[84] Dutz S., Hergt R. (2014). Magnetic Particle Hyperthermia—A Promising Tumour Therapy?. Nanotechnology.

[85] Dutz S., Hergt R., Mürbe J., Töpfer J., Muller R., Zeisberger M., Andrä W., Bellemann M.E. (2006). Magnetic Nanoparticles for Biomedical Heating Applications. Z. Phys. Chem..

[86] Bordelon D.E., Cornejo C., Gruttner C., Westphal F., DeWeese T.L., Ivkov R. (2011). Magnetic Nanoparticle Heating Efficiency Reveals Magneto-Structural Differences when Characterized with Wide Ranging and High Amplitude Alternating Magnetic Fields. J. Appl. Phys..

[87] Meng L., Gan N., Li T., Cao Y., Hu F., Zheng L. (2011). A Three-Dimensional, Magnetic and Electroactive Nanoprobe for Amperometric Determination of Tumor Biomarkers. Int. J. Mol. Sci..

[88] Gonzalez-Fernandez M.A., Torres T.E., Andres-Verges M., Costo R., de la Presa P., Serna C.J., Morales M.R., Marquina C., Ibarra M.R., Goya G.F. (2009). Magnetic nanoparticles for power absorption: Optimizing size, shape and magnetic properties. J. Solid State Chem..

[89] Bae K.H., Park M., Do M.J., Lee N., Ryu J.H., Kim G.W., Kim C., Park T.G., Hyeon T. (2012). Chitosan Oligosaccharide-Stabilized Ferrimagnetic Iron Oxide Nanocubes for Magnetically Modulated Cancer Hyperthermia. Acs Nano.

[90] Nishio K., Ikeda M., Gokon N., Tsubouchi S., Narimatsu H., Mochizuki Y., Sakamoto S., Sandhu A., Abe M., Handa H. (2007). Preparation of size-controlled (30–100 nm) magnetite nanoparticles for biomedical applications. J. Magn. Magn. Mater..

[91] Verges M.A., Costo R., Roca A.G., Marco J.F., Goya G.F., Serna C.J., Morales M.P. (2008). Uniform and Water Stable Magnetite Nanoparticles with Diameters Around the Monodomain-Multidomain Limit. J. Phys. D.

[92] Ristic M., Krehula S., Reissner M., Jean M., Hannoyer B., Music S. (2017). Synthesis and properties of precipitated cobalt ferrite nanoparticles. J. Mol. Struct..

[93] Khandhar A.P., Keselman P., Kemp S.J., Ferguson R.M., Goodwill P.W., Conolly S.M., Krishnan K.M. (2017). Evaluation of PEG-coated iron oxide nanoparticles as blood pool tracers for preclinical magnetic particle imaging. Nanoscale.

[94] Ludwig R., Stapf M., Dutz S., Müller R., Teichgräber U., Hilger I. (2014). Structural Properties of Magnetic Nanoparticles Determine Their Heating Behavior—An Estimation of the In Vivo Heating Potential. Nanoscale Res. Lett..

[95] Hergt R., Dutz S., Müller R., Zeisberger M. (2006). Magnetic Particle Hyperthermia: Nanoparticle Magnetism and Materials Development for Cancer Therapy. J. Phys. Condens. Matter.

[96] Lee J.-H., Jang J.-T., Choi J.-S., Moon S.H., Noh S.-H., Kim J.-W., Kim J.-G., Kim I.-S., Park K.I., Cheon J. (2011). Exchange-Coupled Magnetic Nanoparticles for Efficient Heat Induction. Nat. Nanotechnol..

[97] Lottini E., Lopez-Ortega A., Bertoni G., Turner S., Meledina M., Van Tendeloo G., Fernandez C.D.J., Sangregorio C. (2016). Strongly Exchange Coupled CorelShell Nanoparticles with High Magnetic Anisotropy: A Strategy toward Rare-Earth-Free Permanent Magnets. Chem. Mater..

[98] Phadatare M.R., Meshram J.V., Gurav K.V., Kim J.H., Pawar S.H. (2016). Enhancement of Specific Absorption Rate by Exchange Coupling of the Core-Shell Structure of Magnetic Nanoparticles for Magnetic Hyperthermia. J. Phys. D.

[99] Zhang Q., Castellanos-Rubio I., Munshi R., Orue I., Pelaz B., Gries K.I., Parak W.J., del Pino P., Pralle A. (2015). Model Driven Optimization of Magnetic Anisotropy of Exchange-Coupled Core-Shell Ferrite Nanoparticles for Maximal Hysteretic Loss. Chem. Mater..

[100] Barón M., Hellwich K.H., Hess M., Horie K., Jenkins A.D., Jones R.G., Kahovec J., Kratochvíl P., Metanomski W.V., Mormann W. (2009). Glossary of Class Names of Polymers Based on Chemical Structure and Molecular Architecture (Iupac Recommendations 2009). Pure Appl. Chem..

[101] Laschewsky A. (2014). Structures and Synthesis of Zwitterionic Polymers. Polymers.

[102] Branch D.W., Wheeler B.C., Brewer G.J., Leckband D.E. (2001). Long-term stability of grafted polyethylene glycol surfaces for use with microstamped substrates in neuronal cell culture. Biomaterials.

[103] Sharma S., Johnson R.W., Desai T.A. (2004). Evaluation of the Stability of Nonfouling Ultrathin Poly(ethylene glycol) Films for Silicon-Based Microdevices. Langmuir.

[104] Yang W., Xue H., Li W., Zhang J., Jiang S. (2009). Pursuing “Zero” Protein Adsorption of Poly(carboxybetaine) from Undiluted Blood Serum and Plasma. Langmuir.

[105] Lalani R., Liu L. (2012). Electrospun Zwitterionic Poly(Sulfobetaine Methacrylate) for Nonadherent, Superabsorbent, and Antimicrobial Wound Dressing Applications. Biomacromolecules.

[106] Zhang Z., Chen S., Jiang S. (2006). Dual-Functional Biomimetic Materials:  Nonfouling Poly(carboxybetaine) with Active Functional Groups for Protein Immobilization. Biomacromolecules.

[107] Jiang S., Cao Z. (2010). Ultralow-Fouling, Functionalizable, and Hydrolyzable Zwitterionic Materials and Their Derivatives for Biological Applications. Adv. Mater..

[108] Alexis F., Pridgen E., Molnar L.K., Farokhzad O.C. (2008). Factors Affecting the Clearance and Biodistribution of Polymeric Nanoparticles. Mol. Pharm..

[109] Jang S.H., Wientjes M.G., Lu D., Au J.L.-S. (2003). Drug Delivery and Transport to Solid Tumors. Pharm. Res..

[110] Ma H., Hyun J., Stiller P., Chilkoti A. (2004). “Non-Fouling” Oligo(ethylene glycol)-Functionalized Polymer Brushes Synthesized by Surface-Initiated Atom Transfer Radical Polymerization. Adv. Mater..

[111] Li L., Chen S., Zheng J., Ratner B.D., Jiang S. (2005). Protein Adsorption on Oligo(ethylene glycol)-Terminated Alkanethiolate Self-Assembled Monolayers:  The Molecular Basis for Nonfouling Behavior. J. Phys. Chem. B.

[112] Knop K., Hoogenboom R., Fischer D., Schubert U.S. (2010). Poly(ethylene glycol) in Drug Delivery: Pros and Cons as Well as Potential Alternatives. Angew. Chem. Int. Ed..

[113] Singer S.J., Nicolson G.L. (1972). The Fluid Mosaic Model of the Structure of Cell Membranes. Science.

[114] Zhou Z., Wang L., Chi X., Bao J., Yang L., Zhao W., Chen Z., Wang X., Chen X., Gao J. (2013). Engineered Iron-Oxide-Based Nanoparticles as Enhanced *T*_1_ Contrast Agents for Efficient Tumor Imaging. ACS Nano.

[115] Wang J., Yuan S., Zhang Y., Wu W., Hu Y., Jiang X. (2016). The effects of poly(zwitterions)s *versus* poly(ethylene glycol) surface coatings on the biodistribution of protein nanoparticles. Biomater. Sci..

[116] Muro E., Fragola A., Pons T., Lequeux N., Ioannou A., Skourides P., Dubertret B. (2012). Comparing Intracellular Stability and Targeting of Sulfobetaine Quantum Dots with Other Surface Chemistries in Live Cells. Small.

[117] Mayhew E., Harlos J.P., Juliano R.L. (1973). The effect of polycations on cell membrane stability and transport processes. J. Membr. Biol..

[118] Lowe A.B., McCormick C.L. (2002). Synthesis and Solution Properties of Zwitterionic Polymers. Chem. Rev..

[119] Kudaibergenov S., Jäger W., Laschewsky A. (2006). Polymeric Betaines: Synthesis, Characterization, and Application. Adv. Polym. Sci..

[120] Singh P.K., Singh V.K., Singh M. (2007). Zwitterionic Polyelectrolytes: A Review. e-Polymers.

[121] Tarannum N., Singh M. (2013). Advances in synthesis of Sulfo and Carbo Analogues of Polybetaines: A Review. Rev. Adv. Sci. Eng..

[122] Ostermayer B., Albrecht O., Vogt W. (1986). Polymerizable Lipid Analogs of Diacetylenic Phosphonic-Acids—Synthesis, Spreading Behavior and Polymerization at the Gas-Water Interface. Chem. Phys. Lipids.

[123] Hamaide T., Germanaud L., Leperchec P. (1986). New Polymeric Phosphonatobetaine, Phosphinatobetaine and Carboxybetaine. 1. Syntheses and Characterization by Ir Spectroscopy. Makromol. Chem..

[124] Al-Hamouz O.C.S., Ali S.A. (2012). pH-responsive polyphosphonates using butler’s cyclopolymerization. J. Polym. Sci. Polym. Chem..

[125] Xue C.H., Cai F.F., Liu H.Y. (2008). Ultrasensitive fluorescent responses of water-soluble, zwitterionic, boronic acid-bearing, regioregular head-to-tail polythiophene to biological species. Chem. Eur. J..

[126] Yoshizawa M., Hirao M., Ito-Akita K., Ohno H. (2001). Ion conduction in zwitterionic-type molten salts and their polymers. J. Mater. Chem..

[127] Stöber W., Fink A., Bohn E. (1968). Controlled growth of monodisperse silica spheres in the micron size range. J. Colloid Interface Sci..

[128] Urena-Benavides E.E., Lin E.L., Foster E.L., Xue Z., Ortiz M.R., Fei Y., Larsen E.S., Kmetz A.A., Lyon B.A., Moaseri E. (2016). Low Adsorption of Magnetite Nanoparticles with Uniform Polyelectrolyte Coatings in Concentrated Brine on Model Silica and Sandstone. Ind. Eng. Chem. Res..

[129] Bach L.G., Islam M.R., Kim J.T., Seo S., Lim K.T. (2012). Encapsulation of Fe_3_O_4_ magnetic nanoparticles with poly(methyl methacrylate) *via* surface functionalized thiol-lactam initiated radical polymerization. Appl. Surf. Sci..

[130] Zhang X.A., Lin W., Chen S., Xu H., Gu H. (2011). Development of a Stable Dual Functional Coating with Low Non-specific Protein Adsorption and High Sensitivity for New Superparamagnetic Nanospheres. Langmuir.

[131] Chen Y., Xiong Z., Zhang L., Zhao J., Zhang Q., Peng L., Zhang W., Ye M., Zou H. (2015). Facile synthesis of zwitterionic polymer-coated core-shell magnetic nanoparticles for highly specific capture of N-linked glycopeptides. Nanoscale.

[132] Von der Lühe M., Günther U., Weidner A., Grafe C., Clement J.H., Dutz S., Schacher F.H. (2015). SPION@polydehydroalanine hybrid particles. RSC Adv..

[133] Pombo-Garcia K., Weiss S., Zarschler K., Ang C.-S., Hübler R., Pufe J., Meister S., Seidel J., Pietzsch J., Spiccia L. (2016). Zwitterionic Polymer-Coated Ultrasmall SuperparamagneticIron Oxide Nanoparticles with low Protein Interaction and High Biocompatibility. ChemNanoMat.

[134] Mincheva R., Stoilova O., Penchev H., Ruskov T., Spirov I., Manolova N., Rashkov I. (2008). Synthesis of Polymer-Stabilized Magnetic Nanoparticles and Fabrication of Nanocomposite Fibers Thereof Using Electrospinning. Eur. Polym. J..

[135] Zhang L., Xue H., Gao C., Carr L., Wang J., Chu B., Jiang S. (2010). Imaging and Cell Targeting Characteristics of Magnetic Nanoparticles Modified by a Functionalizable Zwitterionic Polymer with Adhesive 3,4-Dihydroxyphenyl-l-Alanine Linkages. Biomaterials.

[136] Monteil C., Bar N., Bee A., Villemin D. (2016). An Efficient Recyclable Magnetic Material for the Selective Removal of Organic Pollutants. Beilstein J. Nanotechnol..

[137] Korpany K.V., Majewski D.D., Chiu C.T., Cross S.N., Blum A.S. (2017). Iron Oxide Surface Chemistry: Effect of Chemical Structure on Binding in Benzoic Acid and Catechol Derivatives. Langmuir.

[138] Yabu H., Koike R., Hirai D.Y. (2017). Preparation of Poly(Vinyl Catechol-*block*-Styrene) (PVCa-*b*-PSt) Stabilized Iron Oxide Nanoparticles by Ligand Exchange and Janus Particle Formation. J. Nanosci. Nanotechnol..

[139] Li P., Xiao W., Chevallier P., Biswas D., Ottenwaelder X., Fortin M.-A., Oh J.K. (2016). Extremely Small Iron Oxide Nanoparticles Stabilized with Catechol-Functionalized Multidentate Block Copolymer for Enhanced MRI. ChemistrySelect.

[140] Amstad E., Gehring A.U., Fischer H., Nagaiyanallur V.V., Hähner G., Textor M., Reimhult E. (2011). Influence of Electronegative Substituents on the Binding Affinity of Catechol-Derived Anchors to Fe_3_O_4_ Nanoparticles. J. Phys. Chem. C.

[141] Miles W.C., Huffstetler P.P., Goff J.D., Chen A.Y., Riffle J.S., Davis R.M. (2011). Design of stable polyether-magnetite complexes in aqueous media: Effects of the anchor group, molecular weight, and chain density. Langmuir.

[142] Goff J.D., Huffstetler P.P., Miles W.C., Pothayee N., Reinholz C.M., Ball S., Davis R.M., Riffle J.S. (2009). Novel Phosphonate-Functional Poly(ethylene oxide)-Magnetite Nanoparticles Form Stable Colloidal Dispersions in Phosphate-Buffered Saline. Chem. Mater..

[143] Maliakal A., Katz H., Cotts P.M., Subramoney S., Mirau P. (2005). Inorganic Oxide Core, Polymer Shell Nanocomposite as a High *K* Gate Dielectric for Flexible Electronics Applications. J. Am. Chem. Soc..

[144] Caruso F. (2001). Nanoengineering of Particle Surfaces. Adv. Mater..

[145] Babu K., Dhamodharan R. (2009). Synthesis of Polymer Grafted Magnetite Nanoparticle with the Highest Grafting Density via Controlled Radical Polymerization. Nanoscale Res. Lett..

[146] Hassan P.A., Rana S., Verma G. (2015). Making Sense of Brownian Motion: Colloid Characterization by Dynamic Light Scattering. Langmuir.

[147] Lu X.-Y., Wu D.-C., Li Z.-J., Chen G.-Q. (2011). Polymer Nanoparticles. Prog. Mol. Biol. Transl. Sci..

[148] Lim J., Yeap S.P., Che H.X., Low S.C. (2013). Characterization of magnetic nanoparticle by dynamic light scattering. Nanoscale Res. Lett..

[149] Mestrom L., Lenders J.J.M., de Groot R., Hooghoudt T., Sommerdijk N.A.J.M., Artigas M.V. (2015). Stable ferrofluids of magnetite nanoparticles in hydrophobic ionic liquids. Nanotechnology.

[150] Wang F., Zhang X., Liu Y., Lin Z.Y., Liu B., Liu J. (2016). Profiling Metal Oxides with Lipids: Magnetic Liposomal Nanoparticles Displaying DNA and Proteins. Angew. Chem. Int. Ed..

[151] Bonnaud C., Monnier C.A., Demurtas D., Jud C., Vanhecke D., Montet X., Hovius R., Lattuada M., Rothen-Rutishauser B., Petri-Fink A. (2014). Insertion of Nanoparticle Clusters into Vesicle Bilayers. ACS Nano.

[152] Hofmann D., Tenzer S., Bannwarth M.B., Messerschmidt C., Glaser S.-F., Schild H., Landfester K., Mailaender V. (2014). Mass Spectrometry and Imaging Analysis of Nanoparticle-Containing Vesicles Provide a Mechanistic Insight into Cellular Trafficking. ACS Nano.

[153] Clogston J.D., Patri A.K., McNeil S.E. (2011). Zeta Potential Measurement. Characterization of Nanoparticles Intended for Drug Delivery.

[154] Monopoli M.P., Aberg C., Salvati A., Dawson K.A. (2012). Biomolecular Coronas Provide the Biological Identity of Nanosized Materials. Nat. Nanotechnol..

[155] López J., González F., Bonilla F., Zambrano G., Gomez M. (2010). Synthesis and characterization of Fe_3_O_4_ magnetic nanofluid. Rev. LatinAm. Metal. Mat..

[156] Mansfield E., Tyner K.M., Poling C.M., Blacklock J.L. (2014). Determination of Nanoparticle Surface Coatings and Nanoparticle Purity Using Microscale Thermogravimetric Analysis. Anal. Chem..

[157] Liu S., Han Y., Qiao R., Zeng J., Jia Q., Wang Y., Gao M. (2010). Investigations on the Interactions between Plasma Proteins and Magnetic Iron Oxide Nanoparticles with Different Surface Modifications. J. Phys. Chem. C.

[158] Qin L., Xu Y., Han H., Liu M., Chen K., Wang S., Wang J., Xu J., Li L., Guo X. (2015). β-Lactoglobulin (BLG) binding to highly charged cationic polymer-grafted magnetic nanoparticles: Effect of ionic strength. J. Colloid Interface Sci..

[159] Zhao T., Chen K., Gu H. (2013). Investigations on the Interactions of Proteins with Polyampholyte-Coated Magnetite Nanoparticles. J. Phys. Chem. B.

[160] Joseph D., Sachar S., Kishore N., Chandra S. (2015). Mechanistic insights into the interactions of magnetic nanoparticles with bovine serum albumin in presence of surfactants. Colloids Surf. B Biointerfaces.

[161] Alfrey T., Fuoss R.M., Morawetz H., Pinner H. (1952). Amphoteric Polyelectrolytes. II. Copolymers of Methacrylic Acid and Diethylaminoethyl Methacrylate^1^. J. Am. Chem. Soc..

[162] Alfrey T., Morawetz H., Fitzgerald E.B., Fuoss R.M. (1950). Synthetic electrical analog of Proteins^1^. J. Am. Chem. Soc..

[163] Ladenheim H., Morawetz H. (1957). A new type of polyampholyte: Poly(4-vinyl pyridine betaine). J. Polym. Sci..

[164] Billing M., Gräfe C., Saal A., Biehl P., Clement J.H., Dutz S., Weidner S., Schacher F.H. (2017). Zwitterionic Iron Oxide (γ-Fe_2_O_3_) Nanoparticles Based on P(2VP-*grad*-AA) Copolymers. Macromol. Rapid Commun..

[165] Billing M., Schacher F.H. (2016). ATRP of *tert*-Butoxycarbonylaminomethyl acrylate (*t*BAMA): Well-Defined Precursors for Polyelectrolytes of Tunable Charge. Macromolecules.

[166] Yuan J.J., Armes S.P., Takabayashi Y., Prassides K., Leite C.A.P., Galembeck F., Lewis A.L. (2006). Synthesis of Biocompatible Poly[2-(methacryloyloxy)ethyl phosphorylcholine]-Coated Magnetite Nanoparticles. Langmuir.

[167] Hildebrand V., Heydenreich M., Laschewsky A., Möller H.M., Müller-Buschbaum P., Papadakis C.M., Schanzenbach D., Wischerhoff E. (2017). “Schizophrenic” self-assembly of dual thermoresponsive block copolymers bearing a zwitterionic and a non-ionic hydrophilic block. Polymer.

[168] Zhu A., Yuan L., Dai S. (2008). Preparation of Well-Dispersed Superparamagnetic Iron Oxide Nanoparticles in Aqueous Solution with Biocompatible *N*-Succinyl-*O*-carboxymethylchitosan. J. Phys. Chem. C.

[169] Demillo V.G., Zhu X. (2015). Zwitterionic Amphiphile Coated Magnetofluorescent Nanoparticles—Synthesis, Characterization and Tumor Cell Targeting. J. Mater. Chem. B.

[170] Xiao W., Lin J., Li M., Ma Y., Chen Y., Zhang C., Li D., Gu H. (2012). Prolonged *in vivo* circulation time by zwitterionic modification of magnetite nanoparticles for blood pool contrast agents. Contrast Media Mol. Imaging.

[171] Von der Lühe M., Weidner A., Dutz S., Schacher F.H. (2017). Reversible Electrostatic Adsorption of Polyelectrolytes and Bovine Serum Albumin onto Polyzwitterion-Coated Magnetic Multicore Nanoparticles: Implications for Sensing and Drug Delivery. Appl. Nano Mater..

[172] Yeh C.-H., Chen S.-H., Li D.-T., Lin H.-P., Huang H.-J., Chang C.-I., Shih W.-L., Chern C.-L., Shi F.-K., Hsu J.-L. (2012). Magnetic Bead-Based Hydrophilic Interaction Liquid Chromatography for Glycopeptide Enrichments. J. Chromatogr. A.

[173] Wang C., Wang L., Yang W. (2009). Preparation and Characterization of Functional Inorganic/Organic Composite Microspheres via Electrostatic Interaction. J. Colloid Interface Sci..

[174] Zhao Y., Chen Y., Xiong Z., Sun X., Zhang Q., Gan Y., Zhang L., Zhang W. (2017). Synthesis of Magnetic Zwitterionic-Hydrophilic Material for the Selective Enrichment of N-Linked Glycopeptides. J. Chromatogr. A.

[175] Fidale L.C., Nikolajski M., Rudolph T., Dutz S., Schacher F.H., Heinze T. (2013). Hybrid Fe_3_O_4_@amino Cellulose Nanoparticles in Organic Media—Heterogeneous Ligands for Atom Transfer Radical Polymerizations. J. Colloid Interface Sci..

